# Identification of protein phosphatase 4 catalytic subunit as a Wnt promoting factor in pan-cancer and *Xenopus* early embryogenesis

**DOI:** 10.1038/s41598-023-35719-y

**Published:** 2023-06-23

**Authors:** YiLi Wang, WonHee Han, SeokMin Yun, JinKwan Han

**Affiliations:** 1 Laboratory of Developmental Biology, Department of Life Sciences, Pohang University of Science and Technology, Pohang, 37673 Korea; 2 Department of Neurology, F. M. Kirby Neurobiology Center, Boston Children’s Hospital, Harvard Medical School, Boston, MA 02115 USA

**Keywords:** Embryogenesis, Cellular signalling networks, Cancer screening

## Abstract

Protein Phosphatase 4 Catalytic Subunit (PPP4C) is an evolutionarily conserved protein involved in multiple biological and pathological events, including embryogenesis, organogenesis, cellular homeostasis, and oncogenesis. However, the detailed mechanisms underlying these processes remain largely unknown. Thus, we investigated the potential correlation between *PPP4C* and biological processes (BPs) and canonical Wnt signaling using pan-cancer analysis and *Xenopus laevis* (*X. laevis*) embryo model. Our results indicate that *PPP4C* is a potential biomarker for specific cancer types due to its high diagnostic accuracy and significant prognostic correlation. Furthermore, in multiple cancer types, *PPP4C*-related differentially expressed genes (DEGs) were significantly enriched in pattern specification, morphogenesis, and canonical Wnt activation. Consistently, perturbation of Ppp4c in *X. laevis* embryos interfered with normal embryogenesis and canonical Wnt responses. Moreover, biochemical analysis of *X. laevis* embryos demonstrated that both endogenous and exogenous Ppp4c negatively regulated AXIN1 (Wnt inhibitor) abundance. This study provides novel insights into *PPP4C* roles in pattern specification and Wnt activation. The similarities in BPs and Wnt signaling regulation regarding *PPP4C* support the intrinsic link between tumorigenesis and early embryogenesis.

## Introduction

In gene regulatory networks, reversible phosphorylation is a highly dynamic protein modification that incorporates signals from specific pathway(s) and modulates target pathway(s), including itself, without de novo protein synthesis^1^. Thus, dephosphorylation of serine/threonine residues, by protein phosphatase (PPM and PPP) families, regulates multiple signaling pathways and cellular processes. The highly conserved protein phosphatase 4 (PP4) complex, belonging to the type 2A PPP family, is composed of one catalytic subunit (PPP4C) and five regulatory subunits (PPP4R1, PPP4R2, PPP4R3$\upalpha$, PPP4R3$\upbeta$, and PPP4R4). The PP4 complex exists in heterodimeric (PPP4C/PPP4R1; PPP4C/PPP4R4) or heterotrimeric (PPP4C/PPP4R2/PPP4R3$\upalpha$; PPP4C/PPP4R2/PPP4R3$\upbeta$) forms. Its catalytic activity is contributed by PPP4C, while the substrate specificity is mainly determined by the binding regulatory subunits^2–4^. PP4 is reported to regulate multiple cellular events, such as DNA damage response, tumorigenesis, cell migration, immune response, stem cell development, and glucose metabolism^5,6^. The fundamental function of PPP4C in embryogenesis is evidenced by the lethality and dorsal-ventral (DV) patterning defects in mouse and zebrafish PPP4C-deficient embryos, respectively^7,8^. In tumorigenesis, *PPP4C* levels are associated with metastasis and poor prognosis in breast, lung, pancreatic, and colon cancer^9–15^. However, the detailed mechanisms underlying embryogenesis and oncogenesis are still limited.

Canonical Wnt signaling, induced by secreted cysteine-rich Wnt glycoproteins, governs multiple cellular processes, including cell cycle, proliferation, cell fate determination, and tissue patterning. The destruction complex, composed of AXIN, glycogen synthase kinase 3 (GSK3), casein kinase 1 (CK1), and adenomatous polyposis coli tumor-suppressor protein (APC), regulates the stability and abundance of cytosolic $\upbeta$-catenin, which is intimately associated with canonical Wnt signaling “ON/OFF” status^16^. Wnts are essential for organogenesis and tissue homeostasis in multiple tissues, such as skeleton, intestine, hair follicle, skin, hematopoietic and neural systems^17–19^. Therefore, perturbation of canonical Wnt signaling induces congenital malformations and cancer, given its substantial impact on organ development and tissue homeostasis^17^. Moreover, in multiple cancer types, high mutation frequencies in the Wnt components and regulators highlight its function in tumorigenesis regarding cell maintenance, proliferation, and metastasis^20^. In embryogenesis, the most fundamental role of Wnt is as a morphogen shaping DV and anterior–posterior (AP) axes formation^19^. In 1989, McMahon first reported the *X. laevis* axis duplication induced by mouse *Wnt1* mRNA, providing a rapid and convenient assay for estimating Wnt regulation in vertebrates^21^. Genetic interactions between PPP4C and Wnt signaling have been reported. In *Drosophila*, PP4 enhanced Wnt ligand *wg* transcription, while in mouse embryonic stem cells, it promoted neuronal differentiation through the Wnt/RYK pathway^22,23^.

In this study, we investigated *PPP4C*-related BPs and potential correlation between *PPP4C* and Wnt signaling in human pan-cancer RNA-seq profiles and *X. laevis* model. In both species RNA-seq data, *PPP4C* was ubiquitously transcribed in all adult tissues, with relatively lower levels in muscle, brain, heart, and pancreas tissues. In *X. laevis* embryos, *ppp4c* was enriched in the neural crest and head regions during neurula and tailbud stages, respectively. In the cohort study of The Cancer Genome Atlas (TCGA) and Genotype-Tissue Expression (GTEx), *PPP4C* exhibited transcriptional imbalance between normal and tumor tissues in 28 cancer types, with high diagnostic accuracy in 14 and certain accuracy in the remaining. Additionally, *PPP4C* levels were significantly associated with overall survival (OS) in 15 cancer types. Gene Ontology (GO) enrichment analysis of *PPP4C*-related DEGs in pan-cancer revealed that *PPP4C* is linked with pattern specification, morphogenesis, and tissue development, which are essential for embryogenesis. Consistently, overexpression of Ppp4c in *X. laevis* embryos interfered with AP patterning, and loss of Ppp4c impaired anterior structures and melanocytes. Gene Set Enrichment Analysis (GSEA) of the DEGs presented a significant correlation between *PPP4C* and canonical Wnt activation in nine tumor types. This positive correlation was validated in *X. laevis* model, as Wnt responses were compromised in Ppp4c knock-down (Ppp4c-KD) embryos. Additionally, both endogenous and exogenous Ppp4c inhibited AXIN1 abundance, indicating that Ppp4c promotes canonical Wnt signaling at the destruction complex level. Our results highlight the biomarker role of *PPP4C* in multiple cancers, provide novel insights into its function in pattern specification and Wnt signaling regulation, and support the intrinsic link between embryogenesis and oncogenesis.

## Results

### Highly conserved PPP4C is expressed ubiquitously among vertebrates

**Figure 1:**
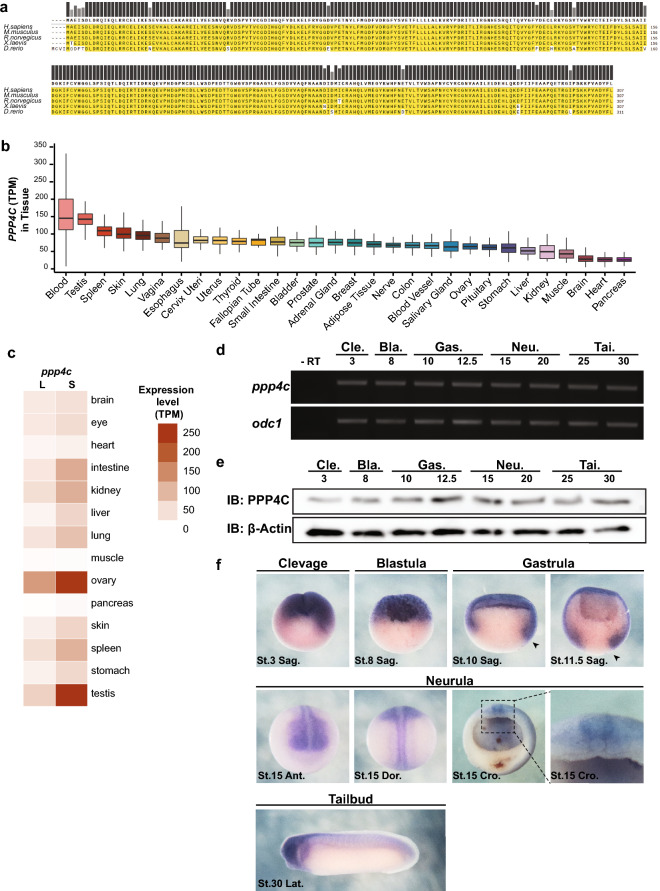
The transcription and expression feature of highly conserved gene *PPP4C* in vertebrates. (**a**) AA sequence alignment of PPP4C from human (*H. sapiens*), mouse (*M. musculus*), rat (*R. norvegicus*), frog (*X. laevis*), and zebrafish (*D. rerio*) species. (**b**) Human *PPP4C* transcription levels in tissues. Boxplot presents the minimum value, the first and third quartile, the median, and the maximum value in each indicated tissue. (**c**) Heatmap of *ppp4c* transcripts in *X. laevis* tissues. This image is generated in Xenbase (www.xenbase.org). (**d**) RT-PCR analysis of *X. laevis* *ppp4c* in embryos from indicated stages. n = 5. *-RT* without reverse transcriptase, *odc1* is loading control. (**e**) Western blot analysis of *X. laevis* Ppp4c in embryos from indicated stages. n = 5. $\upbeta$-Actin is loading control. (**f**) WMISH of *X. laevis* *ppp4c* in embryos from indicated stages. n = 15. *Cle*. cleavage stage, *Bla*. blastula stage, *Gas*. gastrula stage, *Neu*. neurula stage, *Tai*. Tailbud stage, *Sag*. sagittal section, *Ant*. anterior, *Dor*. dorsal, *Cro*. cross section, *Lat*. lateral, *St*. Stage; , dorsal lip.

Amino acid (AA) sequence alignment in Fig. 1a reveals that PPP4C is highly conserved among vertebrate species. Specifically, *X. laevis* Ppp4c exhibits 99% identity with PPP4C in human (NP_001290432.1), mouse (NP_001347393.1), and rat (NP_599186.1) species. The expression pattern of *PPP4C* was explored in adult human and *X. laevis* transcriptome profiles and *X. laevis* embryos. Human single-cell and tissue RNA-seq analyses from The Human Protein Atlas (HPA) and GTEx showed that *PPP4C* was detected in all cell types and tissues, with higher transcriptional levels in reproductive, blood, and skin systems, and lower in muscle, brain, heart, and pancreas (Fig. 1b, Fig. S1). Likewise, *X. laevis* *ppp4c* were highly transcribed in the reproductive systems and lowly in muscle, pancreas, heart, and brain tissues (Fig. 1c), indicating its transcriptional conservatism among vertebrate species. *X. laevis* embryonic *ppp4c* expression was explored by RT-PCR, western blot, and whole-mount in situ hybridization (WMISH). Both the RNA and protein of *ppp4c* were ubiquitously detected in all embryonic stages (Fig. 1d,e). Notably, Ppp4c protein was slightly elevated between late gastrula and mid neurula stages (Fig. 1e). WMISH in Fig. 1f presents the *ppp4c* spatial information. Maternal *ppp4c* transcripts were localized in the animal hemisphere during cleavage (stage 3) and blastula (stage 8) stages. During gastrula stages (stage 10 and 11.5), the *ppp4c*-positive region extended to the dorsal and ventral marginal zone. In the neurula stage (stage 15), *ppp4c* was detected in the anterior part of neural plate and the neural plate border in the trunk region. As development proceeded to the tailbud stage (stage 32), *ppp4c* was enriched in the head region but almost undetectable in others. The *PPP4C* expression differences observed among different adult tissues and between adult and embryonic stages, together with its structural conservatism, promoted us to question its potential roles in embryogenesis and oncogenesis.

### *PPP4C* is a potential biomarker in pan-cancer

With the cohort study of TCGA and GTEx, we compared *PPP4C* transcription levels between 33 tumor and normal tissues. Except for mesothelioma (MESO) and uveal melanoma (UVM), which lacked normal tissue data, *PPP4C* transcripts were significantly increased in most (27/31) tumor types and decreased in acute myeloid leukemia (LAML), while no statistical difference was observed in kidney chromophobe (KICH), pheochromocytoma and paraganglioma (PCPG), or sarcoma (SARC) (Fig. S2a). Paired comparison of tumor and adjacent non-tumor tissue (n > 3) further confirmed the significant elevation of *PPP4C* in bladder urothelial carcinoma (BLCA), breast invasive carcinoma (BRCA), cholangiocarcinoma (CHOL), colon adenocarcinoma (COAD), esophageal carcinoma (ESCA), head and neck squamous cell carcinoma (HNSC), kidney renal clear cell carcinoma (KIRC), kidney renal papillary cell carcinoma (KIRP), liver hepatocellular carcinoma (LIHC), lung squamous cell carcinoma (LUSC), stomach adenocarcinoma (STAD), and thyroid carcinoma (THCA) (Fig. S2b).

The transcriptional differences between tumor and normal tissues were further supported by the diagnostic index factor receiver operating characteristic (ROC) curve. ROC results in Table 1 display that *PPP4C* levels had high predictive accuracy in 14 tumor types, including BLCA, BRCA, cervical squamous cell carcinoma and endocervical adenocarcinoma (CESC), CHOL, COAD, glioblastoma multiforme (GBM), LAML, brain lower grade glioma (LGG), ovarian serous cystadenocarcinoma (OV), pancreatic adenocarcinoma (PAAD), rectum adenocarcinoma (READ), STAD, uterine corpus endometrial carcinoma (UCES), and uterine carcinosarcoma (USC). *PPP4C* levels had certain diagnostic accuracy in adrenocortical carcinoma (ACC), lymphoid neoplasm diffuse large B-cell lymphoma (DLBC), ESCA, HNSC, KIRC, KIRP, LIHC, lung adenocarcinoma (LUAD), LUSC, prostate adenocarcinoma (PRAD), skin cutaneous melanoma (SKCM), PCPG, THCA, and thymoma (THYM) for tumor diagnoses.

**Table 1:** ROC results of *PPP4C* in pan-cancer.

Disease	AUC	95% CI	Cutoff value	Sensitivity	Specificity
ACC	0.824	0.752–0.897	6.246	0.701	0.945
BLCA	0.903	0.845–0.962	6.576	0.887	0.786
BRCA	0.966	0.957–0.975	6.228	0.913	0.949
CESC	0.986	0.974–0.998	6.395	0.958	1
CHOL	1	1	5.787	1	1
COAD	0.927	0.904–0.949	6.206	0.9	0.857
DLBC	0.896	0.863–0.929	6.65	0.957	0.75
ESCA	0.845	0.813–0.877	6.557	0.593	0.917
GBM	0.989	0.979–0.998	5.916	0.988	0.944
HNSC	0.873	0.83–0.917	6.667	0.79	0.818
KIRC	0.722	0.669–0.776	6.027	0.554	0.82
KIRP	0.876	0.83–0.922	6.057	0.862	0.733
LAML	0.946	0.915–0.977	6.683	0.919	0.929
LGG	0.934	0.923–0.945	5.214	0.969	0.806
LIHC	0.848	0.814–0.883	5.852	0.685	0.875
LUAD	0.855	0.83–0.88	6.396	0.767	0.841
LUSC	0.895	0.873–0.916	6.535	0.767	0.929
OV	0.988	0.98–0.997	5.903	0.963	0.977
PAAD	0.983	0.969–0.997	5.553	0.95	0.971
PRAD	0.777	0.734–0.819	5.907	0.875	0.572
READ	0.951	0.926–0.977	6.095	0.903	0.89
SKCM	0.768	0.738–0.798	6.407	0.693	0.801
STAD	0.907	0.884–0.93	6.115	0.739	0.914
TGCT	0.761	0.705–0.817	6.988	0.552	0.952
THCA	0.896	0.874–0.918	6.191	0.756	0.935
THYM	0.776	0.733–0.819	6.611	0.765	0.722
UCES	0.94	0.911–0.969	6.28	0.895	0.95
UCS	0.997	0.993–1	6.29	0.982	0.974

Next, the prognostic potential of *PPP4C* was evaluated by the Kaplan–Meier (KM) estimate in TCGA. The transcription levels of *PPP4C* significantly correlated with patient OS in 15 tumor types, of which *PPP4C* was a risk factor for poor prognosis in ACC, BRCA, HNSC, KIRC, KIRP, LAML, LGG, LIHC, LUAD, MESO, PAAD, and UVM, whereas a protective factor for better OS in CESC, READ, and THYM (Fig. S3). Altogether, the distinct expression pattern between normal and tumor tissues, and the close correlation between *PPP4C* and OS indicate that *PPP4C* is a potential biomarker for tumorigenesis.

**Figure 2:**
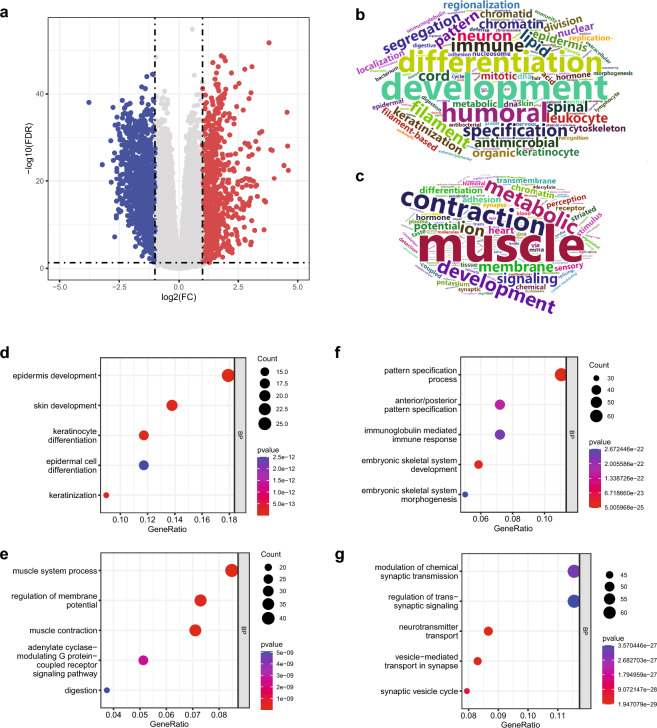
*PPP4C*-related DEGs in pan-cancer are enriched in tissue development, pattern specification, and morphogenesis. (**a**) Volcano plot of DEGs in LGG. Word cloud analysis of GO_BP items of upregulated (**b**) and downregulated (**c**) genes in pan-cancer. GO analysis of upregulated DEGs in ESCA (**d**) and LGG (**f**). GO analysis of downregulated DEGs in ESCA (**e**) and LGG (**g**).

**Figure 3:**
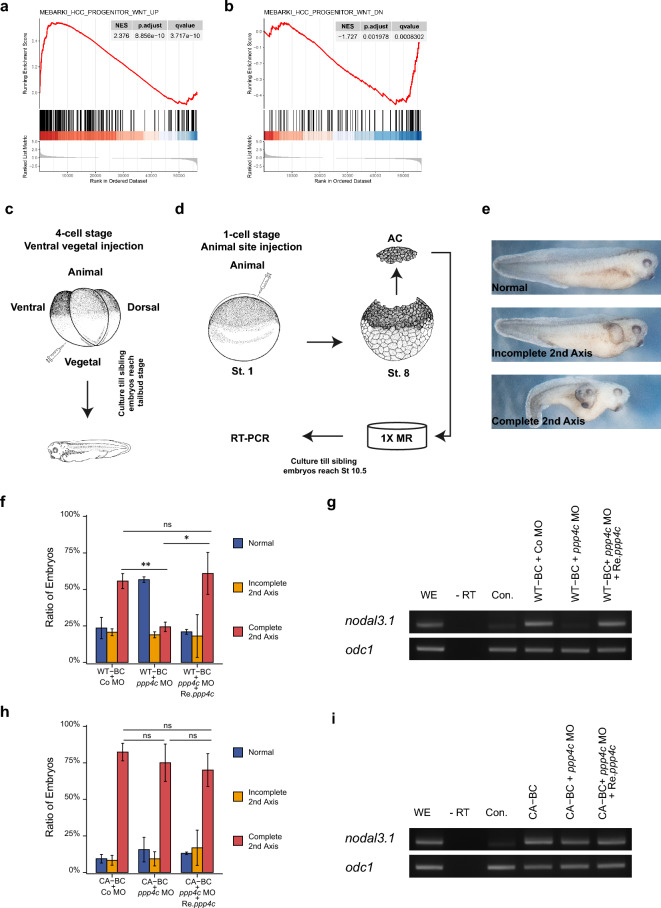
*PPP4C* correlates with canonical Wnt signaling both in tumors and *X. laevis* embryos. Enrichment of genes in the Wnt upregulated (**a**) and downregulated (**b**) gene sets by GSEA in LGG. (**c**) Schematic diagram of axis duplication assay. Embryos were microinjected with indicated reagents at stage 3 and cultured until tailbud stage. (**d**) Schematic diagram of Wnt target gene induction. Embryos were injected at stage 1, AC were dissected at stage 8 and cultured till the sibling embryos reached stage 10.5. n = 30. (**e**) Representative phenotype of (**c**). Embryo images in (**c,d**) were downloaded from Xenbase website^48^. (**f,h**) Quantitative results of axis duplication by indicated reagents ns, *p* ≥ 0.05; *p < 0.05; **p < 0.01. Student t-test. (**g,i**) RT-PCR analysis of animal cap tissue microinjected with indicated reagents. *odc1* was loading control. *WE* whole embryo, *-RT* without reverse transcriptase, *Con*. uninjected control. Reagents used in (**f**–**i**) WT *CTNNB1* mRNA (WT-BC), 50 pg; CA *CTNNB1* mRNA (CA-BC), 20 pg; Co MO, 30 ng; *ppp4c* MO, 30 ng; Re.*ppp4c* mRNA, 250 pg.

**Figure 4:**
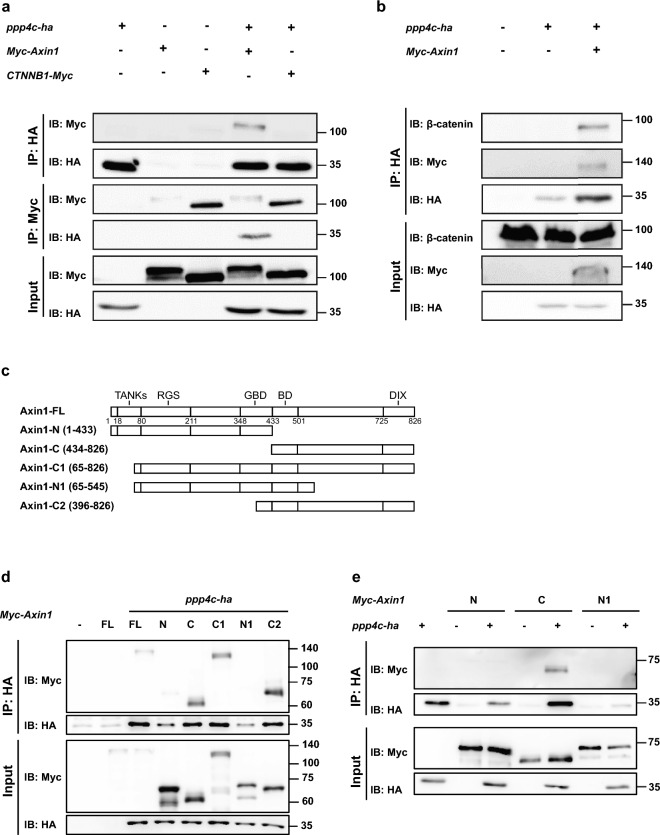
Ppp4c interacts with exogenous AXIN1 in *X. laevis* embryo model. (**a,b**) Co-IP analysis of Ppp4c with AXIN1 and $\upbeta$-catenin in *X. laevis* embryonic AC tissue. AC tissue was dissected at stage 8 and cultured till sibling embryos reached stage 11.5. (**c**) Schematic structure of Myc-tagged truncated AXIN1 mutants. (**d,e**) Co-IP analysis of Ppp4c with various AXIN1 mutants in *X. laevis* whole embryos microinjected with indicated mRNA and harvested at stage 11.5. (**a,b,d,e**) Embryos were microinjected with indicated reagents (1 ng for each mRNA) at 1-cell stage. n = 50. The membranes were cropped at indicated region specified in Supplementary Information.

**Figure 5:**
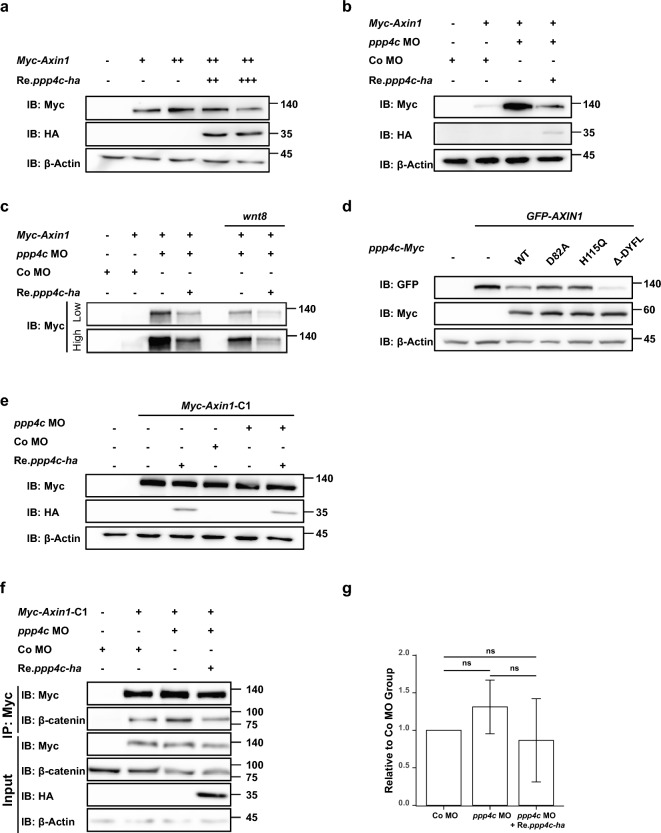
Ppp4c inhibits AXIN1 abundance in *X. laevis* embryo model. (**a–e**) Western blot analysis of whole embryo lysate microinjected with indicated reagents. Embryos were microinjected at stage 1 and harvested at stage 11.5. n = 10. Reagents used in (**a**) +, 1 ng; (**b**) *ppp4c* MO, 60 ng; Co MO, 60 ng; *Myc-Axin1* mRNA, 2 ng; Re.*ppp4c-ha* mRNA, 2 ng; (**c**) *ppp4c* MO, 60 ng; Co MO, 60 ng; *Myc-Axin1* mRNA, 2 ng; Re.*ppp4c-ha* mRNA, 2 ng; *wnt8* mRNA, 100 pg; (**d**) *GFP-AXIN1* mRNA, 2 ng; *ppp4c-Myc* mRNA (WT, D82A, H115Q, $\Delta$-DYFL) 2 ng. (**e**) *Myc-Axin1*-C1 mRNA, 1 ng; *ppp4c-ha* mRNA, 2 ng; *ppp4c* MO, 60 ng; Co MO, 60 ng. (**f**) Co-IP analysis of Myc-AXIN1-C1 with $\upbeta$-catenin in *X. laevis* embryos. Embryos were microinjected with indicated reagents at stage 1 and harvested at stage 11.5. n = 50. *ppp4c* MO, 60 ng; Co MO, 60 ng; *Myc-Axin1*-C1 mRNA, 1 ng; *ppp4c-ha* mRNA, 2 ng. (**g**) Quantitative result of (**f**) measured by ImageJ. N = 3. Values are presented as mean ± standard deviation (SD). ns, *p* ≥ 0.05. Student t-test. (**a,b,d,e,f**) The membranes were cropped at indicated region specified in Supplementary Information.

**Figure 6:**
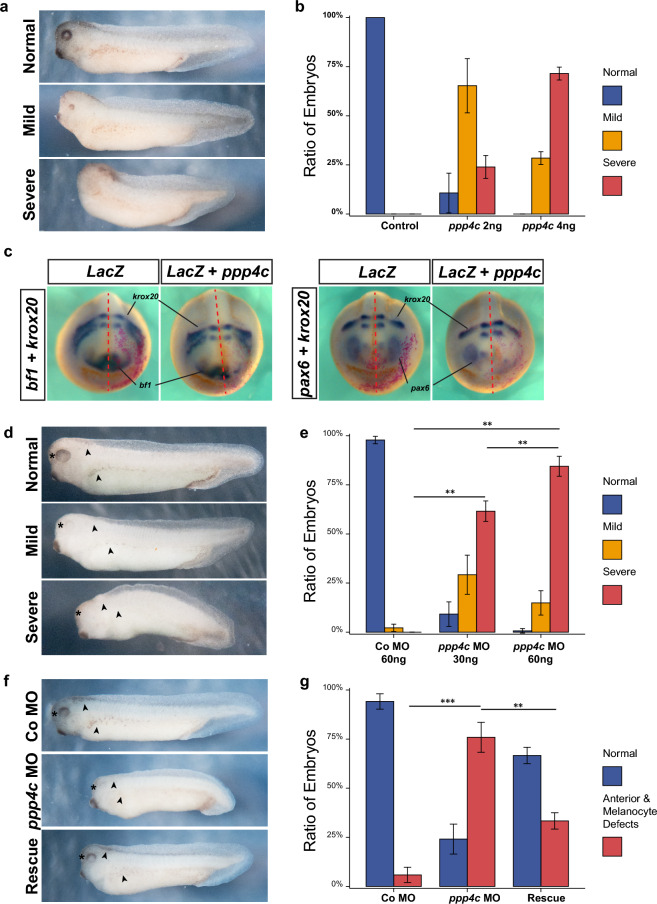
Perturbation of Ppp4c interferes with normal *X. laevis* embryogenesis. Phenotype (**a**) and quantitative data (**b**) of Ppp4c overexpression in *X. laevis* embryos. Embryos were injected with *ppp4c* mRNA in dorsal animal site at stage 3 and cultured into tailbud stage. N = 3. Values are presented as mean ± standard deviation (SD). ‘Mild’ phenotype, reduction of eye and cement gland; ‘Severe’ phenotype, loss of head structure. (**c**) WMISH of Ppp4c overexpression embryos with indicated probes. Red color marks the injection site. *ppp4c* mRNA, 2 ng; *lacZ* mRNA, 250 pg. Phenotype (**d**) and quantitative data (**e**) of *ppp4c* MO in *X. laevis* embryos. Embryos were injected with *ppp4c* MO in animal region at stage 1 and cultured into tailbud stage. N = 3. Values are presented as mean ± standard deviation (SD). ‘Mild’ phenotype, reduction of RPE or melanocytes; ‘Severe’ phenotype, reduction of head size, and complete loss of RPE and melanocytes. Phenotype (**f**) and quantitative data (**g**) of Ppp4c-KD and rescue in *X. laevis* embryos. Embryos were injected with indicated reagents in animal region at stage 1 and cultured into tailbud stage. Co MO, 60 ng; *ppp4c* MO, 60 ng; Rescue, *ppp4c* MO 60 ng + Re.*ppp4c* mRNA 500 pg. N = 3. Values are presented as mean ± standard deviation (SD). ‘Anterior and Melanocyte Defects’ phenotype, reduction of head size, and complete loss of RPE and melanocytes. **p < 0.01; ***p < 0.001. Student t-test. , dorsal and lateral melanocytes; , RPE.

### *PPP4C*-related DEGs are involved in multiple BPs

To uncover *PPP4C*-related BPs in tumorigenesis, we analyzed the DEGs between *PPP4C* high (50%–100%) and low (0%–50%) expression tissues in each tumor. The criteria and numbers of DEGs selected for GO analysis in each tumor are listed in Table S1. The volcano plot of DEGs in LGG is shown in Fig. 2a. Interestingly, besides immune or DNA damage responses that facilitate or impede oncogenesis, *PPP4C*-related BPs focused on regionalization, pattern specification, morphogenesis, metabolism, and the development of skin, keratinocyte, epidermis, and muscle tissues (Fig. 2b,c). Notably, in BLCA, BRCA, CHOL, COAD, ESCA, LGG, MESO, PCPG, PRAD, SARC, THCA, and UCES, elevation of *PPP4C* correlated with activation of genes involved in the skin, epidermis, or keratinocyte-related processes (Fig. 2b,d, Supplementary Dataset File 1). In BLCA, BRCA, CESC, CHOL, DLBC, ESCA, GBM, HNSC, KICH, KIRP, LAML, LIHC, LUSC, MESO, OV, PCPG, PRAD, SARC, STAD, TGCT, THYM, and UCES, the DEGs that negatively correlated with *PPP4C* were enriched in muscle-related processes (Fig. 2c,e, Supplementary Dataset File 2). *PPP4C* levels in ACC, CHOL, GBM, KICH, KIRC, LAML, LGG, LIHC, LUSC, MESO, PCPG, SARC, STAD, THCA, UCES, and UVM positively correlated with pattern specification, regionalization, and morphogenesis (Fig. 2b,f, Supplementary Dataset File 3), which are crucial for early embryogenesis. Besides, in 26 out of 33 tumor types, either high or low, *PPP4C* were significantly correlated with synaptic or synapse-related DEGs (Fig. 2g, Supplementary Dataset File 4, 5).

### *PPP4C* correlates with canonical Wnt signaling in tumors and *X. laevis* embryos

Given the fundamental function of Wnt in AP and DV pattern specification, and the correlation between *PPP4C* and pattern specification in certain tumor types, we explored the relationship between *PPP4C* and canonical Wnt activation through GSEA of DEGs in these tumors. We observed a positive correlation between *PPP4C* and canonical Wnt target genes at the transcriptional level in ACC, CHOL, LAML, LGG, LIHC, PCPG, SARC, THCA, and UVM (Fig. 3a, Supplementary Dataset File 6). Additionally, the Wnt-down gene set was negatively associated with *PPP4C* levels in CHOL and LGG (Fig. 3b, Supplementary Dataset File 6). While in GBM, a negative relationship between *PPP4C* and canonical Wnt activation was observed (Supplementary Dataset File 6).

Furthermore, we validated the correlation between Ppp4c and pattern specification, morphogenesis, and canonical Wnt signaling in the *X. laevis* embryo model, a fast and easy in vitro culture system. In this model, activation of canonical Wnt signaling in the ventral vegetal region or nearly totipotent animal cap (AC) region by ectopic $\upbeta$-catenin induced axis duplication or target gene transcription, respectively (Fig. 3c–g). KD of Ppp4c in *X. laevis* embryos was achieved through injection of *ppp4c* morpholino (*ppp4c* MO), which was designed to block *ppp4c* translation by binding to its 5$'$ untranslated region (5$'$ UTR). The effectiveness and specificity of MO were confirmed by its ability to inhibit the translation of 5$'$ UTR containing *ppp4c-ha* (5$'$ UTR-*ppp4c-ha*) rather than MO-resistant non-5$'$ UTR containing *ppp4c-ha* (*ppp4c-ha* or Re.*ppp4c-ha*) (Fig. S4a,b). In Ppp4c-deficient embryos, the Wnt responses induced by wild-type $\upbeta$-catenin (WT-$\upbeta$-catenin or WT-BC) were compromised but could be rescued by co-injection of Re.*ppp4c* (Fig. 3e–g).

To further identify which hierarchy level in Wnt signaling was affected by Ppp4c, we compared the Wnt responses induced by WT-$\upbeta$-catenin with those induced by constitutively active $\upbeta$-catenin (CA-$\upbeta$-catenin) in *X. laevis* embryos. CA-$\upbeta$-catenin (S37A mutant) cannot be fully phosphorylated by GSK-3$\upbeta$ and is therefore less susceptible to proteasome degradation^24^. The titration of CA-$\upbeta$-catenin to a comparable WT-$\upbeta$-catenin dose in axis duplication is shown in Fig. S5. Unlike WT-$\upbeta$-catenin-induced axis duplication and *nodal3.1* transcription, which were reversibly inhibited upon loss of Ppp4c (Fig. 3f,g), both responses induced by CA-$\upbeta$-catenin were not affected by Ppp4c perturbation (Fig. 3h,i). Collectively, our pan-cancer data determined a correlation between *PPP4C* and canonical Wnt activation. In the *X. laevis* embryo model, Ppp4c promoted canonical Wnt responses at or upstream of $\upbeta$-catenin stability regulation.

### *X. laevis* Ppp4c interacts with AXIN1 in vivo

Co-immunoprecipitation (co-IP) assay of Ppp4c-HA with $\upbeta$-catenin and AXIN1 supported previous observations in *X. laevis* embryos. As shown in Fig. 4a, immunoprecipitates of Ppp4c-HA with an anti-HA antibody retrieved Myc-AXIN1, but not $\upbeta$-catenin-Myc in *X. laevis* embryo animal cap tissue. Consistently, we were able to detect Ppp4c-HA in the reciprocal immunoprecipitates of Myc-AXIN1. As the association between Ppp4c-HA and $\upbeta$-catenin was undetectable, we questioned whether Ppp4c regulated endogenous $\upbeta$-catenin through destruction complex. In the presence of Myc-AXIN1, endogenous $\upbeta$-catenin was detected in the immunoprecipitates of Ppp4c-HA (Fig. 4b), suggesting that Ppp4c indirectly associates with $\upbeta$-catenin through AXIN1. Moreover, co-IP of Ppp4c-HA with several truncated Myc-AXIN1 mutants showed that Ppp4c-HA interacted with several AXIN1 mutants lacking N-terminal parts, rather than the ones lacking C-terminal halves (Fig. 4c–e), suggesting that Ppp4c interacts with AXIN1 through the C-terminal halves.

### *X. laevis* Ppp4c antagonizes AXIN1 abundance

Considering that the phosphorylation status of AXIN1 is closely associated with its stability and scaffold function, we postulate that Ppp4c may regulate AXIN1 abundance or scaffold function through dephosphorylation. As the endogenous Axin1 abundance in *X. laevis* embryos is below the western blot detection threshold, we evaluated ectopic Myc-AXIN1 regarding protein abundance and its interaction with endogenous $\upbeta$-catenin in embryos. In the lysate of stage 11 embryos, ectopic Myc-AXIN1 abundance was dose-dependently decreased by co-injection of *ppp4c-ha* mRNA (Fig. 5a), and reversibly accumulated in Ppp4c-deficient embryos (Fig. 5b,c), conveying that Ppp4c inhibits AXIN1 at the protein level. Furthermore, as ectopic Myc-AXIN1 in Ppp4c-KD embryos shifted behind the normal ones (Fig. 5c, lanes 2 and 3), and two catalytic-dead Ppp4c mutants (D82A, H115Q) failed to inhibit Myc-AXIN1 abundance (Fig. 5d, lanes 1–5), AXIN1 inhibition is most likely to be correlated with its protein modification, such as dephosphorylation. Notably, Ppp4c could partially rescue AXIN1 band shift in the presence of Wnt8 (Fig. 5c, Lane 2–4 and 7), suggesting that exogenous Wnt ligand promotes Ppp4c’s function in AXIN1 protein modification. Additionally, we observed that the tail motif deletion Ppp4c mutant ($\Delta$DYFL) expressed enhanced Myc-AXIN1 inhibition compared to WT-Ppp4c (Fig. 5d, lanes 2, 3, and 6), implying that the tail motif may negatively regulate Ppp4c activity in AXIN inhibition during gastrula stages. In the scaffold function study, to normalize precipitated AXIN1 abundance within groups, we utilized a TANKs domain truncated AXIN1 mutant (Myc-AXIN1-C1), which possessed interaction sites with both $\upbeta$-catenin and Ppp4c (Fig. 4c,d), and its abundance was not Ppp4c-dependent (Fig. 5e,f). Perturbation of Ppp4c rarely affected the abundance of endogenous $\upbeta$-catenin that immunoprecipitated with Myc-AXIN1-C1 (Fig. 5f,g). Collectively, these observations indicate that Ppp4c inhibits AXIN1 abundance in a catalytic-dependent manner.

### Perturbation of Ppp4c interferes with normal embryogenesis in *X. laevis*

In pan-cancer study, *PPP4C* levels correlated with multiple BPs, such as regionalization, pattern specification, morphogenesis, metabolism, and the development of skin, keratinocyte, epidermis, and muscle tissues. In both *X. laevis* embryos and certain tumor tissues, *PPP4C* levels significantly correlated with canonical Wnt activation. Therefore, we questioned whether interference with Ppp4c could affect normal embryogenesis. As activation of canonical Wnt signaling in dorsal animal (DA) or ventral vegetal regions of embryos could induce head truncation or axis duplication respectively, we wondered whether excess Ppp4c could generate similar phenotypes. Overexpression of Ppp4c in the DA region, where the future head structure originates from, dose-dependently reduced the eye field, cement gland, and overall head structures in tailbud embryos (Fig. 6a,b). In addition, WMISH of several markers of head structure shows that Ppp4c DA overexpression reduced *bf1* (forebrain) and *pax6* (eye), rather than *krox20* (hindbrain) (Fig. 6c), indicating excess Ppp4c alters AP patterning in embryogenesis. Unlike $\upbeta$-catenin (50 pg), injection of *ppp4c* or *ppp4c*-$\Delta$DYFL (up to 2 ng) failed to induce axis duplication when injected ventral-vegetally (Fig. S6a), implying that Ppp4c enhances rather than directly induces canonical Wnt activation in *X. laevis* embryos and may require the cooperation from other Wnt axis levels.

In KD studies, we first validated the efficiency of injection sites, and the most efficient and consistent KD phenotype was observed in 1-cell stage AC injected embryo (Fig. S6b). Injection of *ppp4c* MO into animal hemisphere of 1-cell stage embryos dose-dependently impaired anterior structures and melanocytes located on dorsal and lateral regions (Fig. 6d,e). The head size, retinal pigment epithelium (RPE), and melanocyte defects in Ppp4c-KD embryos can be partially rescued by co-injection of Re.*ppp4c* mRNA (Fig. 6f,g), suggesting that Ppp4c is essential for proper development of anterior structures and melanocytes during *X. laevis* embryogenesis. Notably, anterior structure defects in Ppp4c-KD embryos differ from those in Ppp4c overexpression embryos. In Ppp4c-KD embryos, the cement gland size was unaffected and anterior structures were not fully eliminated, indicating that the anterior deficiencies in the Ppp4c-KD embryos are more likely caused by tissue-specific rather than global AP patterning defects. Collectively, perturbation of Ppp4c in early embryos interferes with normal embryogenesis.

## Discussion

Like other serine/threonine phosphatases, PP4 participates in multiple cellular processes, including DNA damage response, cell cycle regulation, tumorigenesis, immune response, stem cell pluripotency maintenance, and differentiation^11,25–29^. In human cancer RNA-seq profiles and *X. laevis* embryos, we explored the function of PPP4C, the PP4 catalytic subunit. *PPP4C* levels correlated with pattern specification and canonical Wnt activation in both fields. Specifically, we propose that Ppp4c promotes canonical Wnt activity at the destruction complex level, as *X. laevis* Ppp4c interacted with AXIN1 and catalytically inhibited its protein abundance. Furthermore, anterior structure and melanocyte defects in Ppp4c-deficient *X. laevis* embryos highlight its developmental significance.

Perturbation of certain genes which are active in embryos and silent in adult tissue can tip the balance between proliferation and differentiation, inducing congenital diseases or cancer. Scattered evidence of *PPP4C* elevation has been documented in breast, lung, ovarian, colorectal, and pancreatic ductal tumors^10–14^. In lung cancer and colorectal carcinoma, PPP4C promoted tumorigenesis by facilitating tumor cell survival, proliferation, migration, and invasion^10,13^. In ovarian cancer cells, PP4 inhibition by fostriecin or KD enhanced immune response and reduced homologous recombination in tumor cells, leading to tumor repression^14^. In breast tumor cells, PPP4C can act as either an anti-tumor factor promoting cell apoptosis through PEA15 regulation or an oncogenic factor facilitating proliferation and migration in a cell-content dependent manner^9,30^. However, to our knowledge, no research has addressed its diagnostic or prognostic potential in pan-cancer. Consistent with previous reports, *PPP4C* mRNA levels were significantly upregulated in most cancers (27/31) and decreased in LAML. Moreover, the unbalanced transcriptional levels of *PPP4C* between normal and tumor tissues contributed to its high diagnostic accuracy in 14 tumor types. In tumorigenesis, PPP4C probably behaves as an oncogenic or protective factor cell-content-dependently, as its transcription levels corresponded to different OS outcomes in various cancer types. *PPP4C*-related DEGs in specific tumors were also consistent with the existing roles of PPP4C, as the DEGs closely correlated with immune response and DNA replication-related processes.

Additionally, *PPP4C* levels in multiple cancers were closely associated with various BPs, including morphogenesis, regionalization, and pattern specification. The DV patterning role of Ppp4c has been reported in zebrafish model. Depletion of Ppp4ca or Ppp4cb resulted in embryo dorsalization, as indicated by the expansion of organizer-specific marker *chordin*, and reduction of ventral marker *gata2* at the shield stage^8^. However, injection of *ppp4c* MO into the future dorsal marginal zone of 4-cell stage *X. laevis* embryos, rarely affected the dorsal organizer (*gsc*) domain at the gastrula stage (data not shown). As the *ppp4c* domain was not spatially overlapped with the organizer, we postulated that zygotic Ppp4c is not essential for organizer formation. However, due to MO limitations, we cannot preclude that maternally inherited Ppp4c protein participates in organizer shaping processes. In *X. laevis* model, Ppp4c correlated with AP pattern specification, as overexpression of Ppp4c in DA dose-dependently reduced anterior structures at the tailbud stage. Reduction of forebrain (*bf1*) and eye (*pax6*) markers in neurula embryos, without impact on hindbrain marker (*krox20*), further corroborated its anterior-inhibition effect. The discrepancies between zebrafish and *X. laevis* models may be due to: (1) other phosphatases functional redundancy; (2) protein modifications affecting PP4 complex assembly; and (3) zebrafish and higher vertebrate PPP4C structural differences at N$'$ terminal 9 AA, which may lead to binding regulatory subunits and further substrates differences. The developmental role of Ppp4c in anterior structures and melanocytes was supported by the KD studies, and the *ppp4c* positive domain overlapped with neural crest tissue, which has potential to develop into future sensory organs, melanocytes, ganglia, and facial skeleton structures. Interestingly, DEGs in GBM, UVM, and LAML were significantly enriched in retina, melanosome, or pigment-related BPs (Supplementary Dataset File 4), further supporting the similarities between embryogenesis and tumorigenesis. We also observed that *PPP4C*-related DEGs in specific tumors were involved in epidermis, skin, keratinocyte, and muscle-related BPs. As *PPP4C* was transcribed unevenly between skin and muscle in both human and *X. laevis* tissue RNA-seq profiles, exploring the potential of *PPP4C* in epidermis/muscle cell fate determination in embryonic and adult tissue will benefit future studies. These cellular response similarities imply that the tumors mentioned above may share similar cell content with embryos concerning each biological process, and clues from cancer studies may provide alternative views for embryonic research. Therefore, *X. laevis* embryos may serve as an alternative model for cancer research and vice versa.

PPP4C is reported to regulate Wnt, BMP, and Hedgehog signaling pathways^8,22,31^. We specifically explored the relationship between *PPP4C* and Wnt signaling due to its fundamental function in AP patterning and neural crest formation. The positive genetic interaction between PP4 and Wnt signaling was initially described in *Drosophila* through an RNAi screen^32^. In further studies, PP4 enhanced Notch-driven wnt ligand *wg* transcription or initiated the nuclear translocation of the receptor-like tyrosine kinase (Ryk) intracellular domain to promote Wnt signaling^22,23^. A Wnt inhibition role was reported in embryonic stem cells rather than cancer cells. The PP4 complex promoted histone deacetylation, inhibiting Wnt target gene *brachyury* transcription^25^. In our study, *PPP4C* levels correlated with canonical Wnt activation in multiple cancers, and *X. laevis* Ppp4c was required for canonical Wnt responses in embryos. Overexpression of Ppp4c in the DA region reduced the most anterior structures, phenocopying weak Wnt activation in embryogenesis. Unlike neural crest-derived melanocytes necessitating persistent Wnt for specification, RPE pigment cells originate from the forebrain and require dynamic Wnt regulation. Although we did not observe apparent DV defects by *ppp4c* MO as seen in zebrafish, the BMP regulatory activity may also be required for Ppp4c-mediated anterior structure formation in *X. laevis* embryos. Therefore, the functional time-point, region, and the cooperation between its Wnt and BMP regulation in Ppp4c-mediated RPE and melanocyte development, are yet to be determined in future studies.

AXIN abundance is several magnitudes lower than other components in the destruction complex, thus, regulation of AXIN abundance and scaffold function is essential for canonical Wnt signaling regulation. AXIN phosphorylation, by GSK3 and CK1, stabilizes it and enhances its binding affinity for $\upbeta$-catenin and GSK3 in the destruction complex^33–35^. In Wnt “ON” status, AXIN is dephosphorylated, leading to the dissociation of $\upbeta$-catenin from the destruction complex and subsequent AXIN degradation^36^. Several PP complexes (PP1, PP2A, and PP2C) have been reported to interact with AXIN. PP1 antagonizes CK1 on the phosphorylation status of AXIN, releasing it from GSK3^37^. Moreover, PP1 changes AXIN conformation into “inactivation” mode upon LRP6 phosphorylation, diminishing its binding to phospho-LRP6, leaving it unperturbed for continuous signaling^38^. PP2C decreases AXIN half-life, eliciting a synergistic response with $\upbeta$-catenin and Wnt1 in cells^36^. Another AXIN interactor, PP2A, contributes to Wnt inhibition possibly through dephosphorylation of GSK3$\upbeta$^39^. In this study, Ppp4c was identified as a Wnt-promoting factor in *X. laevis*. Unlike previously reported Wnt-promoting roles, Ppp4c was proposed to target AXIN1 in $\upbeta$-catenin regulation, which was supported by the following evidence: (1) unlike WT-$\upbeta$-catenin, stable CA-$\upbeta$-catenin-induced Wnt responses were Ppp4c perturbation-resistant; (2) AXIN1, particularly the C-terminal halves, co-precipitated with Ppp4c; (3) $\upbeta$-catenin could not be detected in the immunoprecipitates of Ppp4c unless AXIN1 was present; and (4) *X. laevis* Ppp4c inhibited AXIN1 abundance in a catalytic-dependent manner.

AXIN can be subjected to either PARsylation/ubiquitination by Tankyrase/RNF146 or ubiquitination by Smurf2 for proteasome degradation^40–42^. In our study, TANKs domain truncated AXIN1 mutant was resistant to Ppp4c perturbation, suggesting that PARsylation by Tankyrase may be involved in Ppp4c-mediated AXIN1 degradation. Interestingly, DYFL motif deletion in Ppp4c enhanced its AXIN1 inhibition in gastrula embryos. The tail motif, which is highly conserved in PP2A family catalytic subunit factors, is subject to methylation and phosphorylation modification^43–45^. For PP2AC, DYFL methylation or phosphorylation within or upstream was found to differentially affected subunit affinity, thereby tipping the balance between different PP2A complexes^43^. Likewise, demethylation in the PPP4C DYFL motif diminished its interaction with PP4R1, PP4R2, PP4R3$\upalpha$, and PP4R3$\upbeta$^45^. Considering that the substrate specificity is mainly determined by binding subunits and the DYFL motif modifications affect PP complex assemble, we postulate that DYFL modification during the gastrula stage is unfavorable for AXIN-inhibitory PP4 complex formation. Notably, Ppp4c or Ppp4c-$\Delta$DYFL failed to induce axis duplication when injected ventral-vegetally. Based on the existing literature and our data, we postulate that this may be due to three possibilities as below: (1) abundance limitation of the PP4 regulatory subunits or other co-factors. In ventral vegetal overexpression studies, these proteins may be the limiting factors for the formation of Wnt-promoting PP4 machinery. In this case, insufficient co-factors or PP4 complex may compromise the Wnt responses in the ventral vegetal region when excess Ppp4c present. (2) BMP inhibition. In C2C12 cells, PPP4C was found to interact with Smad1 without affecting its linker or C-terminal phosphorylation, and the nuclear translocation of p-Smad1 shuttled PPP4C from the cytosol to the nucleus^8^. Therefore, in the ventral region, the accumulation of p-Smad1 and its nuclear translocation upon BMP activation may favor the nuclear rather than cytosolic PP4 complex formation, reduce the interaction between Ppp4c and AXIN1, and ultimately affect the Wnt regulatory activity of Ppp4c. (3) Canonical Wnt activity requirements. Ppp4c could partially rescue AXIN1 band shift in KD embryos with exogenous Wnt8. However, endogenous Wnt activity is not completely blocked in *X. laevis* embryos, therefore, whether Wnt activation is required for Ppp4c-mediated AXIN1 inhibition, and if Ppp4c dephosphorylates other Wnt signaling components to degrade AXIN, are yet to be determined.

These findings collectively provide a novel Ppp4c function in regulating canonical Wnt signaling. However, this research had some limitations due to technical issues. In embryo model, as massive amounts of $\upbeta$-catenin exist on the plasma membrane, perturbation of cytosolic $\upbeta$-catenin, by loss or gain of Ppp4c, was not sensitively detected by western blot. Additionally, in our unpublished data, excess of Wnt8 enhanced the AXIN1 inhibitory activity of Ppp4c in *Xenopus* embryos. In future investigations, it would be fascinating to investigate the following: (1) whether Ppp4c promotes canonical Wnt signaling through endogenous Axin1 or not; (2) if so, whether this mechanism can be applied to the tumors identified in pan-cancer analysis; (3) the binding regulatory subunit(s) for Wnt activation and the PP4 assembly regulation; (4) if and how Ppp4c dephosphorylation correlates with Wnt/Tankyrase/RNF146-mediated AXIN degradation; and (5) whether Ppp4c regulates other Wnt axis levels and how these regulations interact. A detailed analysis will uncover the precise mechanism of Ppp4c in canonical Wnt signaling and the involvement of other pathways in this regulation.

## Materials and methods

### Data acquisition

The RNA-seq data of normal human tissues [GTEx Analysis V8 (dbGaP Accession phs000424.v8.p2)] and single cell were downloaded from GTEx (https://www.gtexportal.org/home/datasets) and HPA (https://www.proteinatlas.org/about/download) respectively. The expression feature of *PPP4C* in pan-cancer and its diagnostic value were explored in a combined cohort study of TCGA and GTEx downloaded from UCSC Xena (https://xenabrowser.net/datapages/). The analysis of prognostic value, DEGs, GO enrichment, and GSEA were performed in the transcriptome data across 33 tumor types downloaded from the TCGA (https://portal.gdc.cancer.gov) together with the relevant clinical profile.

### Diagnostic and prognostic value analysis

The diagnostic potential of *PPP4C* in each cancer type was assessed by the ROC curve in the cohort of TCGA and GTEx data. The accuracy of prediction was presented by the area under the curve (AUC), which was calculated by the package pROC (v 1.18.0). AUC values were classified as high accuracy (> 0.9), certain accuracy (> 0.7 and $\le$ 0.9), and low accuracy (> 0.5 and $\le$ 0.7).

KM plots were used to investigate the association between *PPP4C* level and patients OS in pan-cancer. The cutoff value for *PPP4C* high and low expression groups was determined using the minimum *p* value method^46^. To avoid extreme imbalance in group numbers, the cutoff value in each cancer was restricted between the upper and lower quartile of *PPP4C* values. The survminer package (v 0.4.9) was used for visualization, and the survival package (v 3.4-0) was used for statistical analysis. *p* < 0.05 was considered as statistically significant.

### GO enrichment analysis and GSEA of the DEGs

DEGs were analyzed using the DESeq2 package (v 1.36.0) based on the gene counts data between *PPP4C* high and low groups (divided by the median). The minimal values of |log2FoldChange| and numbers of DEGs (*p*.adjust < 0.05) in each tumor type selected for GO enrichment analysis are listed in Table S1. GO enrichment analysis of the DEGs was conducted using the clusterProfiler package (v 4.4.4). In each tumor, the top 25 GO biological process items with a *p*.adjust value < 0.05 were collected for word cloud analysis using the wordcloud2 (v 0.2.1) and jiebaR (0.11) packages.

GSEA was performed on DEGs to determine the correlation between canonical Wnt activation and *PPP4C* level. The clusterProfiler package was used for statistical analysis, and the enrichplot package (v 1.16.1) was used for visualization. The C2 curated gene sets (c2.all.v7.5.symbols.gmt) in the MSigDB Collection were used as the reference gene collection. *p*.adjust value < 0.05, false discovery rate (qvalue) < 0.25, and normalized enrichment score (NES) > 1 were considered significant enrichment.

### Embryos manipulation and microinjection

*X. laevis* eggs were obtained, fertilized, and staged as previously described^47,48^. Embryos were cultured in 0.33$\times$Modified Ringer (MR) until the mid-blastula transition, after which transferred to 0.1$\times$MR until harvest. Embryo injection was performed using HARVARD Injector in 0.33$\times$MR containing 4% Ficoll-PM400 (GE Healthcare) at indicated stages. Injected embryos were cultured in 4% Ficoll-400 in 0.33$\times$MR before the mid-blastula transition. For the axis duplication assay, mRNA or morpholino was injected into the ventral vegetal region at the 4-cell stage, and the embryos were cultured until the tailbud stage (Fig. 3c)^49^. In the animal cap explant induction experiment, animal caps were dissected at stage 8 and cultured until the sibling embryos reached stage 10.5 (Fig. 3d)^49^.

### Plasmid constructs, mRNA synthesis, and MO

The open reading frame of *ppp4c* was amplified by PCR from stage 9 *X. laevis* cDNA using Q5$^{\circledR }$ Hot Start High-Fidelity DNA Polymerase (NEB), cloned into pGEM$^{\circledR }$-T Easy Vector (Promega), and subcloned into the *BamHI* and *XbaI* sites of pCS2+, pCS2+HA, and pCS2+6Myc vectors. Full length of mouse *Axin1* and human *AXIN1* constructs were kindly provided by Dr. Frank Costantini. The $\upbeta$-catenin *CTNNB1* constructs were obtained as described previously^50^. Point and deletion mutagenesis were used to generate catalytic-dead (D82A, H115Q) and deletion ($\Delta$DYFL) mutants of *ppp4c*, and the truncated forms (1–433, 434–826, 65–826, 65–545, and 396–826) of mouse *Axin1*. Capped mRNA was synthesized from the linearized constructs using the SP6 mMessage mMachine kit (Ambion) following the manufacturer’s instructions. Antisense MO for *X. laevis* *ppp4c* and control MO were obtained from Gene Tools, LLC, with the sequences 5$'$-CATGGTCCCTCCTCAATGAGCCTGT-3$'$ and 5$'$-CCTCTTACCTCAGTTACAATTTATA-3$'$, respectively.

### RT-PCR

The target gene abundance was analyzed semi-quantitatively by RT-PCR. 5 or 10 whole embryos or 30 explants were harvested as described and snapped in liquid nitrogen. Total RNA was extracted from the samples using TRIZOL reagent (Invitrogen) according to the manufacturer’s protocol. Purified total RNA (5 $\upmu$g) was reverse transcribed into cDNA using the M-MLV reverse transcriptase (Promega). *odc1* was used as an internal control for normalization. Amplification of each target gene was performed in a 25 $\upmu$l system using Taq polymerase (Solgent). Primers used in RT-PCR are listed in Table S2.

### WMISH and *lacZ*$\upbeta$-galactosidase Staining

WMISH was performed as previously described and stained with NBT/BCIP (Sigma)^51^. The antisense in situ probe labeled with digoxigenin (DIG) against *ppp4c* was transcribed using T7 RNA polymerase with the template generated by linearization of *ppp4c*-T-easy construct with *SpeI*. The probes against *bf1*, *pax6*, and *krox20* were obtained as previously described^52,53^. Indication of injection site was performed by *lacZ* $\upbeta$-galactosidase staining. 250 pg of *lacZ* mRNA was coinjected into the embryos. $\upbeta$-galactosidase-expressing embryos at the indicated stage were fixed in MEMFA solution (10% formaldehyde, 0.1 M MOPS (pH 7.4), 2 mM EGTA, 1 mM $\hbox {MgSO}_{4}$) for 30 min at room temperature after being rinsed with PBS (pH 7.4, 137 mM NaCl, 2.7 mM KCl, 10 mM $\hbox {Na}_{2}\hbox {HPO}_{4}$, 1.8 mM $\hbox {KH}_{2}\hbox {PO}_{4}$). Then, the embryos were stained for 1–2 h at room temperature in PBS containing 5 mM $\hbox {K}_{3}\hbox {Fe(CN)}_{6}$, 5 mM $\hbox {K}_{4}\hbox {Fe(CN)}_{6}$, 1 mg/ml X-gal (red), 2 mM MgCl${}_{2}$. The stained embryos were fixed in MEMFA solution for 4 h for further WMISH.

### Western blot, immunoprecipitation, and antibodies

Western blot assays and co-IP were carried out as previously described^49,54^. Briefly, embryos or animal cap explants were homogenized in Triton-X 100 lysis buffer [20 mM Tris-HCl (pH 7.5), 1% Triton X-100, 140 mM NaCl, 10% glycerol, 1 mM EGTA, 1.5 mM $\hbox {MgCl}_{2}$, 1 mM DTT, 1 mM sodium vanadate ($\hbox {Na}_{3}\hbox {VO}_{4}$), 50 mM NaF, 20 $\upmu$g/ml aprotinin, 40 $\upmu$g/ml leupeptin, and 0.75 mM phenylmethylsulfonyl fluoride (PMSF)] for western blot assay, or IP buffer [20 mM Tris-Cl (pH 7.5), 150 mM NaCl, 2 mM EDTA (pH 7.5), 1% TritonX-100, 1 mM $\hbox {Na}_{3}\hbox {VO}_{4}$, 100 mM NaF, 25 mM $\upbeta$-glycerophosphate, 20 $\upmu$g/ml aprotinin, 40 $\upmu$g/ml leupeptin, and 0.75 mM PMSF] for co-IP assay. The lysate was centrifuged to remove insoluble debris and yolk. In western blot, an equivalent amount of 1/4 whole embryo protein was loaded for each well of the SDS-PAGE gel, and $\upbeta$-Actin was used as the internal control. In co-IP assay, the indicated antibodies were added to the supernatants and incubated at $4\,^{\circ }\hbox {C}$ for 6 h. Rec-Protein G-Sepharose$^{\circledR }$ 4B Conjugate (Invitrogen) was added, and the mixture was incubated at $4^{\circ }\hbox {C}$ overnight. The immune complexes bound to Protein-G beads were washed six times with IP buffer and then subjected to SDS-PAGE gel for western blot. The mobility shift assay was performed on a 6.5% SDS-PAGE gel. The antibodies used in western blot and co-IP were listed in Table S3. Immunoreactivity was visualized by incubation with SuperSignal$^{\textrm{TM}}$ West Femto Maximum Sensitivity Substrate (Thermo) following the manufacturer’s instructions or with ECL solution unless specified. For the blots in western blot that required equal or more than 3 antibodies hybridization, and co-IP experiments, the membranes were cropped at indicated region before antibody hybridization to reduce bleaching times and non-specific staining interference .

### Statistical analyses and image process

Statistical analysis was conducted using R software (v 4.2.1) downloaded from R website (https://www.r-project.org/). The Wilcoxon rank sum test was used to compare the difference of *PPP4C* between normal and tumor tissues in non-paired and paired samples. For the phenotype and western blot analysis, the comparison between groups was assessed by Student’s t-test. The *p* value was calculated using the rstatix package (v 0.7.0). The ggplot2 package (v 3.3.6) was used for visualization unless specified. *p* < 0.05 was considered statistically significant (ns, *p* $\ge$ 0.05; *p < 0.05; **p < 0.01; ***p < 0.001). PPP4C AA sequence blast was performed in SnapGene (v 6.1). The figures were processed and combined using Adobe Photoshop (v 23.2.1) and Adobe Illustrator (v 26.1).

### Ethical approval

This study was approved by the Institutional Animal Care and Use Committee of Pohang University of Science and Technology. All methods and procedures were carried out in accordance with relevant guidelines and regulations. The study is reported in accordance with ARRIVE guidelines.

## Supplementary Information

Supplementary Information 1.

Supplementary Information 2.

Supplementary Information 3.

Supplementary Information 4.

Supplementary Information 5.

Supplementary Information 6.

Supplementary Information 7.

## Data Availability

The datasets generated and analyzed during the current study are available from the corresponding author on reasonable request.

## References

[1] Day EK, Sosale NG, Lazzara MJ (2016). Cell signaling regulation by protein phosphorylation: A multivariate, heterogeneous, and context-dependent process. Curr. Opin. Biotechnol..

[2] Chen GI (2008). Pp4r4/kiaa1622 forms a novel stable cytosolic complex with phosphoprotein phosphatase 4. J. Biol. Chem..

[3] Lee D-H (2010). A pp4 phosphatase complex dephosphorylates rpa2 to facilitate dna repair via homologous recombination. Nat. Struct. Mol. Biol..

[4] Hwang J, Lee JA, Pallas DC (2016). Leucine carboxyl methyltransferase 1 (lcmt-1) methylates protein phosphatase 4 (pp4) and protein phosphatase 6 (pp6) and differentially regulates the stable formation of different pp4 holoenzymes. J. Biol. Chem..

[5] Ramos F, Villoria MT, Alonso-Rodríguez E, Clemente-Blanco A (2019). Role of protein phosphatases pp1, pp2a, pp4 and cdc14 in the dna damage response. Cell Stress.

[6] Park J, Lee D-H (2020). Functional roles of protein phosphatase 4 in multiple aspects of cellular physiology: A friend and a foe. BMB Rep..

[7] Shui J-W, Hu MC-T, Tan T-H (2007). Conditional knockout mice reveal an essential role of protein phosphatase 4 in thymocyte development and pre-t-cell receptor signaling. Mol. Cell. Biol..

[8] Jia S (2012). Protein phosphatase 4 cooperates with smads to promote bmp signaling in dorsoventral patterning of zebrafish embryos. Dev. Cell.

[9] Xie W (2022). Comprehensive analysis of pppcs family reveals the clinical significance of ppp1ca and ppp4c in breast cancer. Bioengineered.

[10] Wang B, Zhu X-X, Pan L-Y, Chen H-F, Shen X-Y (2020). Pp4c facilitates lung cancer proliferation and inhibits apoptosis via activating mapk/erk pathway. Pathol. Res. Pract..

[11] Wang B (2008). Protein phosphatase pp4 is overexpressed in human breast and lung tumors. Cell Res..

[12] Weng S (2012). Overexpression of protein phosphatase 4 correlates with poor prognosis in patients with stage ii pancreatic ductal adenocarcinomapp4 in pancreatic ductal adenocarcinoma. Cancer Epidemiol. Biomark. Prev..

[13] Li X (2015). High expression of protein phosphatase 4 is associated with the aggressive malignant behavior of colorectal carcinoma. Mol. Cancer.

[14] Raja R (2022). Pp4 inhibition sensitizes ovarian cancer to nk cell-mediated cytotoxicity via stat1 activation and inflammatory signaling. J. Immunother. Cancer.

[15] Hang J (2021). The role of phosphoprotein phosphatases catalytic subunit genes in pancreatic cancer. Biosci. Rep..

[16] Clevers H, Nusse R (2012). Wnt/$$\beta$$-catenin signaling and disease. Cell.

[17] Clevers H (2006). Wnt/$$\beta$$-catenin signaling in development and disease. Cell.

[18] Mulligan KA, Cheyette BN (2012). Wnt signaling in vertebrate neural development and function. J. Neuroimmune Pharmacol..

[19] Steinhart Z, Angers S (2018). Wnt signaling in development and tissue homeostasis. Development.

[20] Yang K (2016). The evolving roles of canonical wnt signaling in stem cells and tumorigenesis: Implications in targeted cancer therapies. Lab. Invest..

[21] McMahon AP, Moon RT (1989). Ectopic expression of the proto-oncogene int-1 in xenopus embryos leads to duplication of the embryonic axis. Cell.

[22] Hall ET, Pradhan-Sundd T, Samnani F, Verheyen EM (2017). The protein phosphatase 4 complex promotes the notch pathway and wingless transcription. Biol. Open.

[23] Chang W-H (2017). Smek1/2 is a nuclear chaperone and cofactor for cleaved wnt receptor ryk, regulating cortical neurogenesis. Proc. Natl. Acad. Sci..

[24] Björklund P, Lindberg D, Åkerström G, Westin G (2008). Stabilizing mutation of ctnnb1/beta-catenin and protein accumulation analyzed in a large series of parathyroid tumors of swedish patients. Mol. Cancer.

[25] Lyu J, Jho E-H, Lu W (2011). Smek promotes histone deacetylation to suppress transcription of wnt target gene brachyury in pluripotent embryonic stem cells. Cell Res..

[26] Lyu J (2013). Protein phosphatase 4 and smek complex negatively regulate par3 and promote neuronal differentiation of neural stem/progenitor cells. Cell Rep..

[27] Brechmann M (2012). A pp4 holoenzyme balances physiological and oncogenic nuclear factor-kappa b signaling in t lymphocytes. Immunity.

[28] Lipinszki Z (2015). Centromeric binding and activity of protein phosphatase 4. Nat. Commun..

[29] Villoria MT (2019). Pp4 phosphatase cooperates in recombinational dna repair by enhancing double-strand break end resection. Nucleic Acids Res..

[30] Mohammed HN, Pickard MR, Mourtada-Maarabouni M (2016). The protein phosphatase 4-pea15 axis regulates the survival of breast cancer cells. Cell. Signal..

[31] Liao H (2020). Protein phosphatase 4 promotes hedgehog signaling through dephosphorylation of suppressor of fused. Cell Death Dis..

[32] Swarup S, Pradhan-Sundd T, Verheyen EM (2015). Genome-wide identification of phospho-regulators of wnt signaling in drosophila. Development.

[33] Yamamoto H (1999). Phosphorylation of axin, a wnt signal negative regulator, by glycogen synthase kinase-3$$\beta$$ regulates its stability. J. Biol. Chem..

[34] Jho E-H, Lomvardas S, Costantini F (1999). A gsk3$$\beta$$ phosphorylation site in axin modulates interaction with $$\beta$$-catenin and tcf-mediated gene expression. Biochem. Biophys. Res. Commun..

[35] Tacchelly-Benites O, Wang Z, Yang E, Lee E, Ahmed Y (2013). Toggling a conformational switch in wnt/$$\beta$$-catenin signaling: Regulation of axin phosphorylation: The phosphorylation state of axin controls its scaffold function in two wnt pathway protein complexes. BioEssays.

[36] Strovel ET, Wu D, Sussman DJ (2000). Protein phosphatase 2c$$\alpha$$ dephosphorylates axin and activates lef-1-dependent transcription. J. Biol. Chem..

[37] Luo W (2007). Protein phosphatase 1 regulates assembly and function of the $$\beta$$-catenin degradation complex. EMBO J..

[38] Kim S-E (2013). Wnt stabilization of $$\beta$$-catenin reveals principles for morphogen receptor-scaffold assemblies. Science.

[39] Chu D (2016). Gsk-3$$\beta$$ is dephosphorylated by pp2a in a leu309 methylation-independent manner. J. Alzheimers Dis..

[40] Huang S-MA (2009). Tankyrase inhibition stabilizes axin and antagonizes wnt signalling. Nature.

[41] Zhang Y (2011). Rnf146 is a poly (adp-ribose)-directed e3 ligase that regulates axin degradation and wnt signalling. Nat. Cell Biol..

[42] Kim S, Jho E-H (2010). The protein stability of axin, a negative regulator of wnt signaling, is regulated by smad ubiquitination regulatory factor 2 (smurf2). J. Biol. Chem..

[43] Longin S (2007). Selection of protein phosphatase 2a regulatory subunits is mediated by the c terminus of the catalytic subunit. J. Biol. Chem..

[44] Martin M, Kettmann R, Dequiedt F (2010). Recent insights into protein phosphatase 2a structure and regulation: The reason why pp2a is no longer considered as a lazy passive housekeeping enzyme. Biotechnol. Agron. Soc. Environ..

[45] Lee J, Lee D-H (2014). Leucine methylation of protein phosphatase pp4c at c-terminal is critical for its cellular functions. Biochem. Biophys. Res. Commun..

[46] Mizuno H, Kitada K, Nakai K, Sarai A (2009). Prognoscan: A new database for meta-analysis of the prognostic value of genes. BMC Med. Genom..

[47] Newport J, Kirschner M (1982). A major developmental transition in early xenopus embryos: I. Characterization and timing of cellular changes at the midblastula stage. Cell.

[48] Nieuwkoop, P. D. & Faber, J. Normal table of. *Xenopus laevis* 252 (1994).

[49] Choi S-C, Han J-K (2005). Rap2 is required for wnt/$$\beta$$-catenin signaling pathway in xenopus early development. EMBO J..

[50] Han W, Koo Y, Chaieb L, Keum B-R, Han J-K (2022). Uchl5 controls $$\beta$$-catenin destruction complex function through axin1 regulation. Sci. Rep..

[51] Harland RM (1991). In situ hybridization: An improved whole-mount method for xenopus embryos. Methods Cell Biol..

[52] Kim H (2009). Xenopus wntless and the retromer complex cooperate to regulate xwnt4 secretion. Mol. Cell. Biol..

[53] Lee H (2018). Head formation requires dishevelled degradation that is mediated by march2 in concert with dapper1. Development.

[54] Cheong S-M, Kim H, Han J-K (2009). Identification of a novel negative regulator of activin/nodal signaling in mesendodermal formation of xenopus embryos. J. Biol. Chem..

