# Supplementary material for: Identification of protein phosphatase 4 catalytic subunit as a Wnt promoting factor in pan-cancer and Xenopus early embryogenesis
Source: Sci Rep. 2023 Jun 23;13:10240. doi: 10.1038/s41598-023-35719-y (PMC10290155; doi:10.1038/s41598-023-35719-y)
Supplement: Supplementary file 7 — Supplementary Information 7. [file 41598_2023_35719_MOESM7_ESM.pdf]

Supplementary Information

Identification of Protein Phosphatase 4 Catalytic Subunit as a Wnt Promoting Factor in Pan-cancer and *Xenopus* Early Embryogenesis

YiLi Wang[1], WonHee Han[1,2], SeokMin Yun[1], and JinKwan Han[1,*]

1. Laboratory of Developmental Biology, Department of Life Sciences, Pohang University of Science and Technology, Pohang, 37673, Korea

2. Department of Neurology, F. M. Kirby Neurobiology Center, Boston Children's Hospital, Harvard Medical School, Boston, MA, 02115, USA

* Corresponding author: jkh@postech.ac.kr

Supplementary Figures

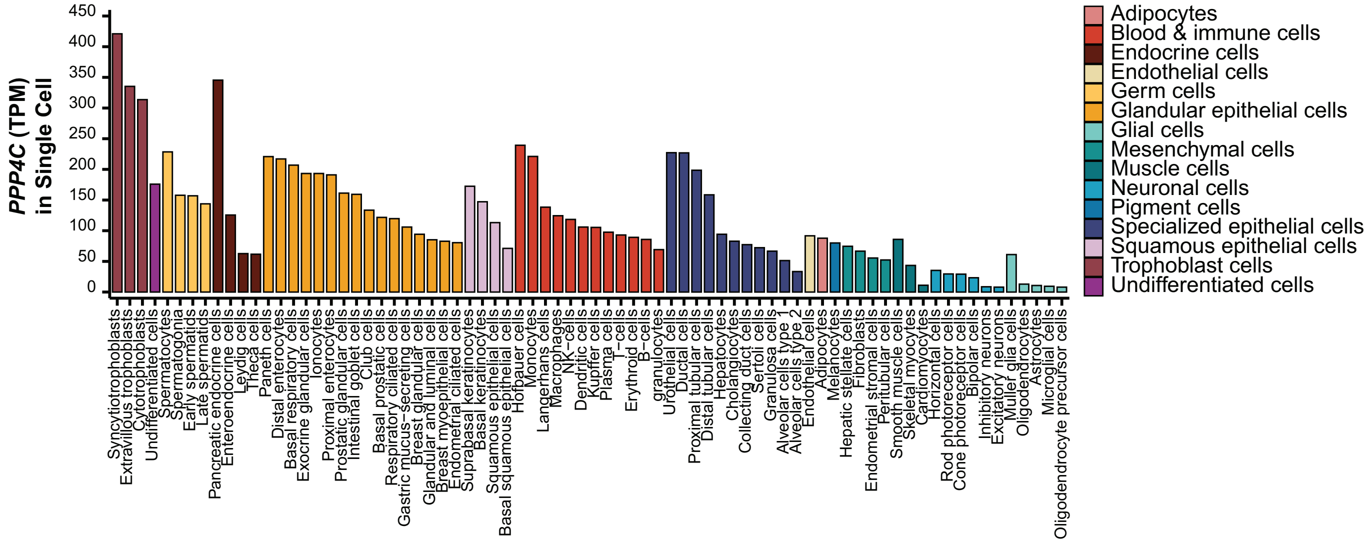

Figure S1. **Human *PPP4C* transcription level in single cells.**

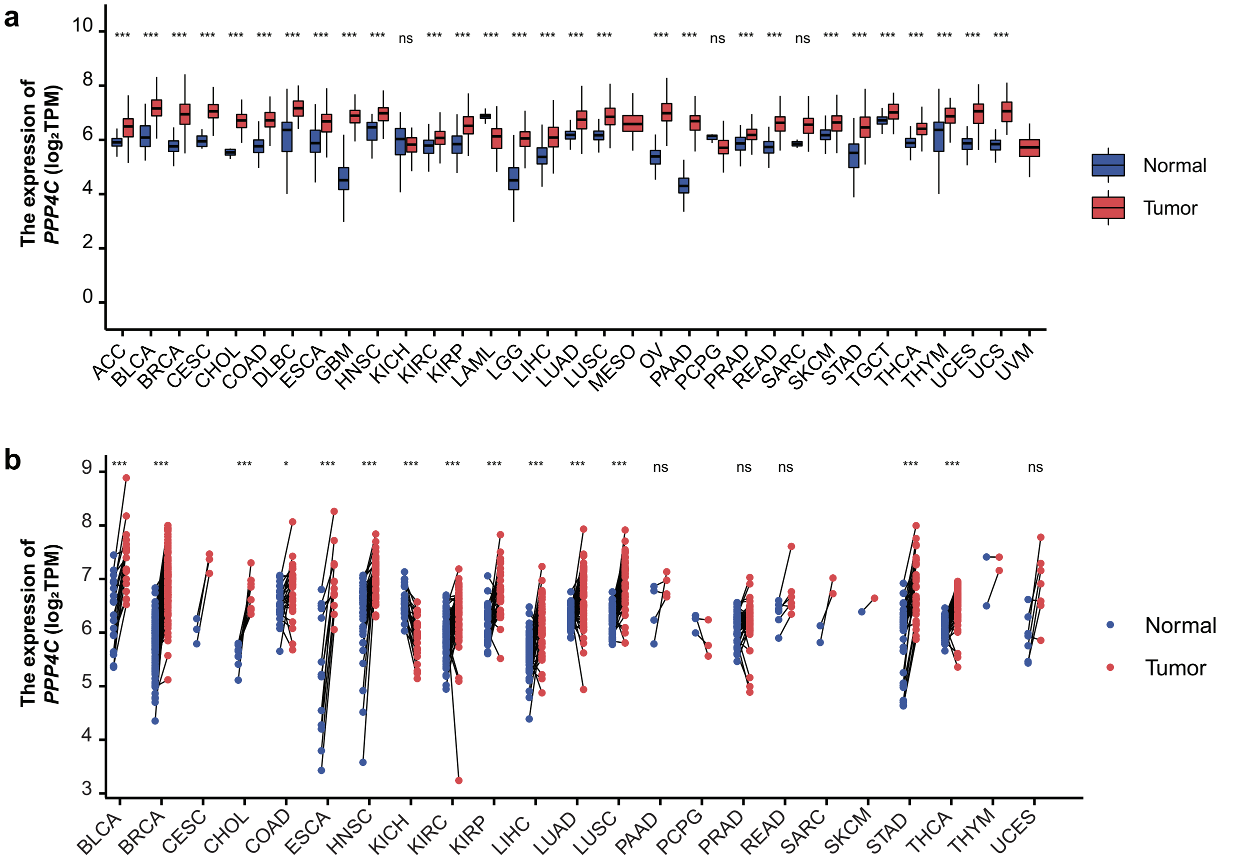

Figure S2. Transcription level of *PPP4C* in tumors and normal tissue. (a) *PPP4C* transcription in tumors and normal tissues with the data from the coherent study of TCGA and GTEx. **(b)** *PPP4C* transcription in TCGA tumors and adjacent normal tissues. ns, $p \geq 0.05$; *, $p < 0.05$; $p < 0.01$; ***, $p < 0.001$. Wilcoxon rank sum test.

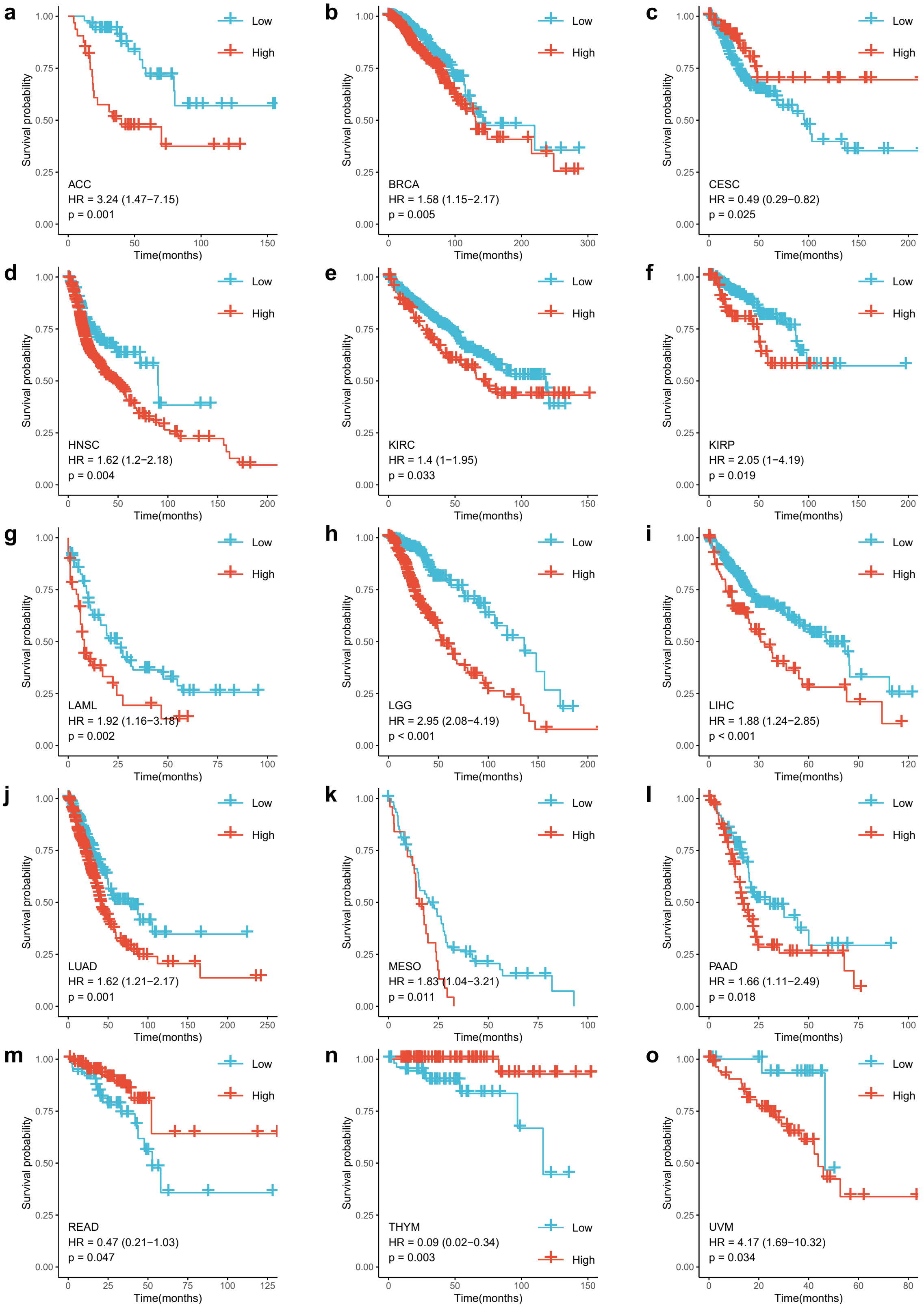

Figure S3. **Correlation between *PPP4C* level and the overall survival prognosis of cancers. (a)** ACC; **(b)** BRCA; **(c)** CESC; **(d)** HNSC; **(e)** KIRC; **(f)** KIRP; **(g)** LAML; **(h)** LGG; **(i)** LIHC; **(j)** LUAD; **(k)** MESO; **(l)** PAAD; **(m)** READ; **(n)** THYM; **(o)** UVM.

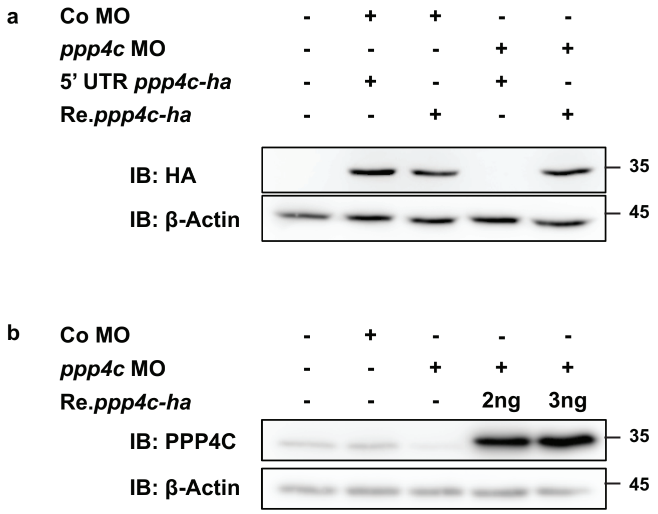

Figure S4. **Efficiency and specificity test of *ppp4c* MO. (a, b)** Western blot of embryos injected with indicated reagent. Embryos were injected into animal region at 1 cell stage and cultured till stage 11. Co MO, 60 ng; *ppp4c* MO, 60 ng; 5′ UTR *ppp4c-ha* mRNA, 1 ng; Re.*ppp4c-ha* mRNA, 1 ng.

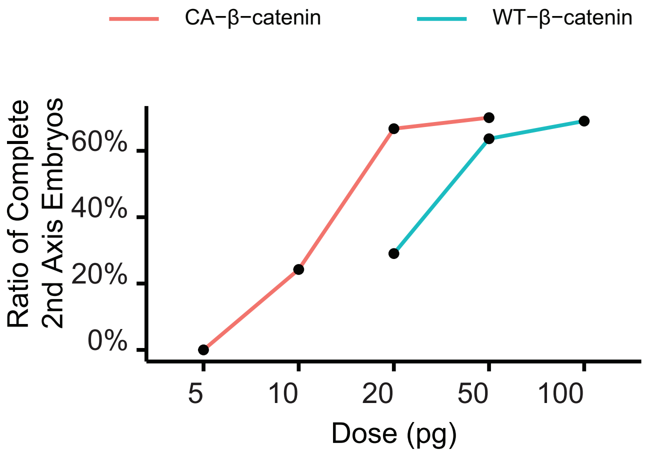

Figure S5. **Titration curve of axis duplication by WT-β-catenin and CA-β-catenin.** Embryos were injected with indicated mRNA at ventral vegetal blastomere at 4 cell stage and cultured till tailbud stage.

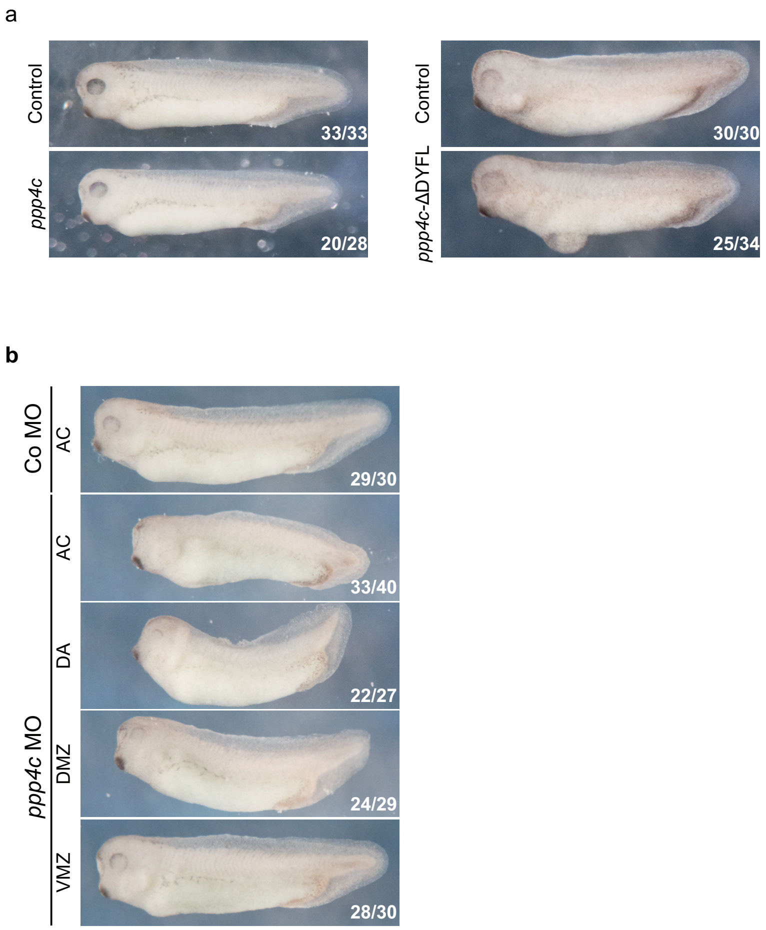

Figure S6. Representative phenotype in overexpression and KD of Ppp4c in *X.laevis*. (a) Overexpression phenotype of Ppp4c and Ppp4c-ΔDYFL in ventral vegetal region of 4-cell embryos. *ppp4c* mRNA, 2 ng; *ppp4c*–ΔDYFL mRNA, 2ng. **(b)** *ppp4c* MO injection sites validation. AC: animal cap, 1-cell stage embryos were injected at animal hemisphere. DA, dorsal animal; DMZ, dorsal marginal zone; VMZ, ventral vegetal zone; DA, DMZ, VMZ, 4-cell stage embryos were injected at indicated sites . co MO, 60 ng; *ppp4c* MO, 60 ng.

Supplementary Tables

Disease	Upregualted		Downregulated	
	$\lvert log_2FC \rvert$	Numbers	$\lvert log_2FC \rvert$	Numbers
ACC	1	200	1	420
BLCA	1	432	1	1198
BRCA	1	354	1	3823
CESC	1	221	1	682
CHOL	1	1124	1	2605
COAD	1	83	1	5404
DLBC	1	262	1	1303
ESCA	1	359	1.5	1283
GBM	1	77	1	1546
KICH	1	142	1	81
KIRC	0.5	611	1	1140
KIRP	0.5	388	1	1425
LAML	1	647	1	1350
LGG	1	907	1	1270
LIHC	1	283	1	1378
LUAD	1	539	1.5	2790
LUSC	1	351	1	991
MESO	1	372	1	759
OV	1	191	1	248
PAAD	1	429	/	/
PCPG	1	526	1	473
PRAD	1	73	1	439
READ	1	142	1	209
SARC	1	910	1	958
SKCM	1	294	1	376
STAD	1	153	1	1029
TGCT	1	463	1	2879
THCA	1	285	1	904
THYM	1	2047	1	5685
UCES	1	367	1	1065
UCS	1	295	1	137
UVM	1	476	1	1799

Table S1. Threshold and numbers of DEGs in pan-cancer for GO analysis

Gene Symbol	Forward Primer(5′-3′)	Reverse Primer(5′-3′)
ppp4c	ATGACTGAAATCAGTGACCTC	ATCTGATCCAGTGTCTGGAT
nodal3.1	CGAGTGCAAGAAGGTGGACA	ATCTTCATGGGGACACAGGA
odc1	CAGCTAGCTGTGGTGTGG	CAACATGGAAACTCACACC

Table S2. Primer sequence for RT-PCR

Antibody	Species	Dilution	Company	Catalog
β-catenin	Rabbit	1:10000	Santa Cruz Biotechnology	sc-7199
β-Actin	Mouse	1:4000	Santa Cruz Biotechnology	sc-47778
GFP	Mouse	1:1000	Santa Cruz Biotechnology	sc-9996
HA	Mouse	1:1000	Santa Cruz Biotechnology	sc-7392
c-Myc	Mouse	1:2000	Santa Cruz Biotechnology	sc-40
c-Myc	Rabbit	1:2000	Santa Cruz Biotechnology	sc-789
PPX	Mouse	1:1000	Santa Cruz Biotechnology	sc-374106
Anti-Rabbit IgG	Goat	1:10000	Sigma Aldrich Co.	A0545
Anti-Mouse IgG	Goat	1:10000	Thermo Scientific	NCI1430KR

Table S3. Antibodies used in Western blot and co-IP

WB and PCR original data

Figure 1

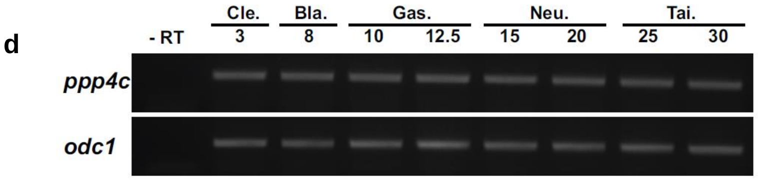

ppp4c

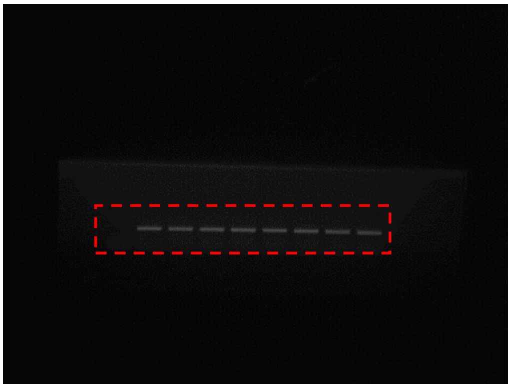

odc1

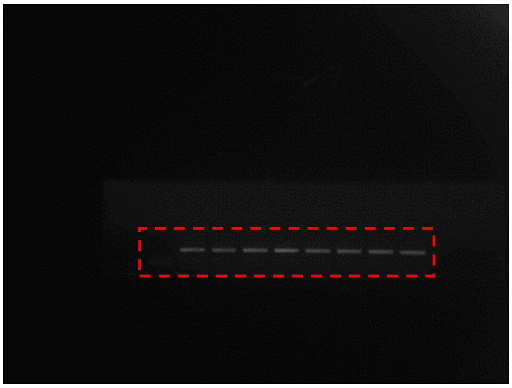

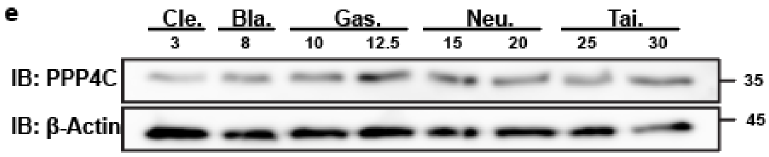

Ppp4c

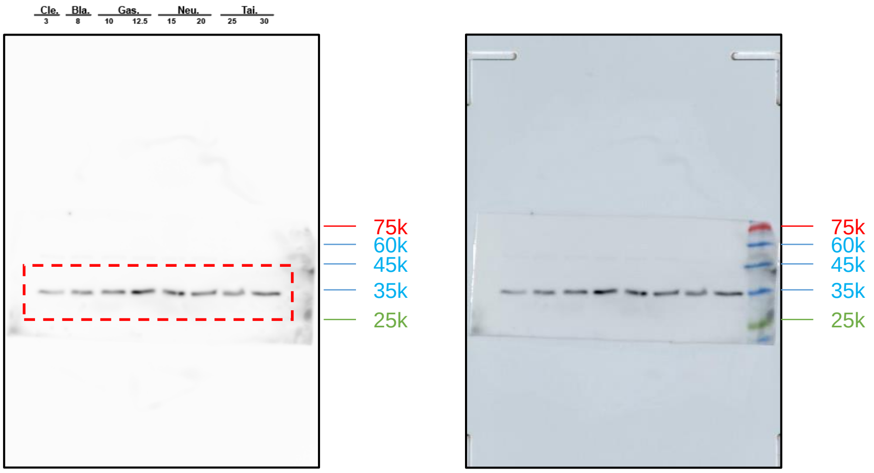

β-Actin

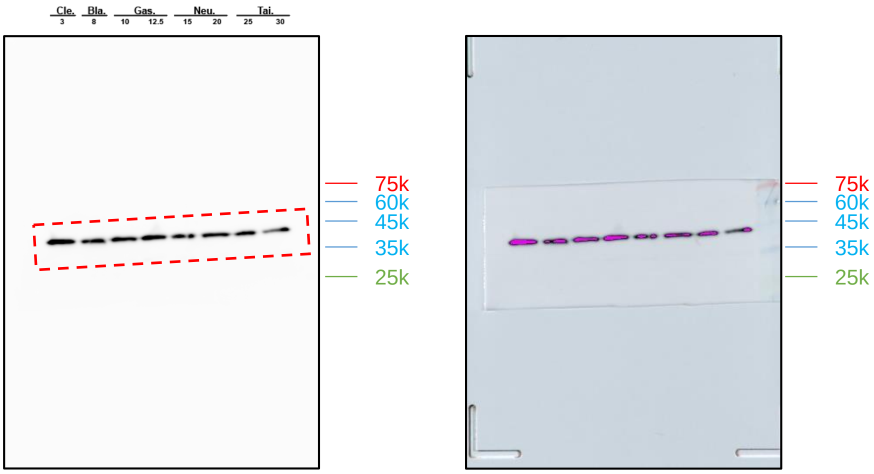

Figure 1e: same membrane, exposure: HA 30 s, β-Actin: 20 s.

Figure 3

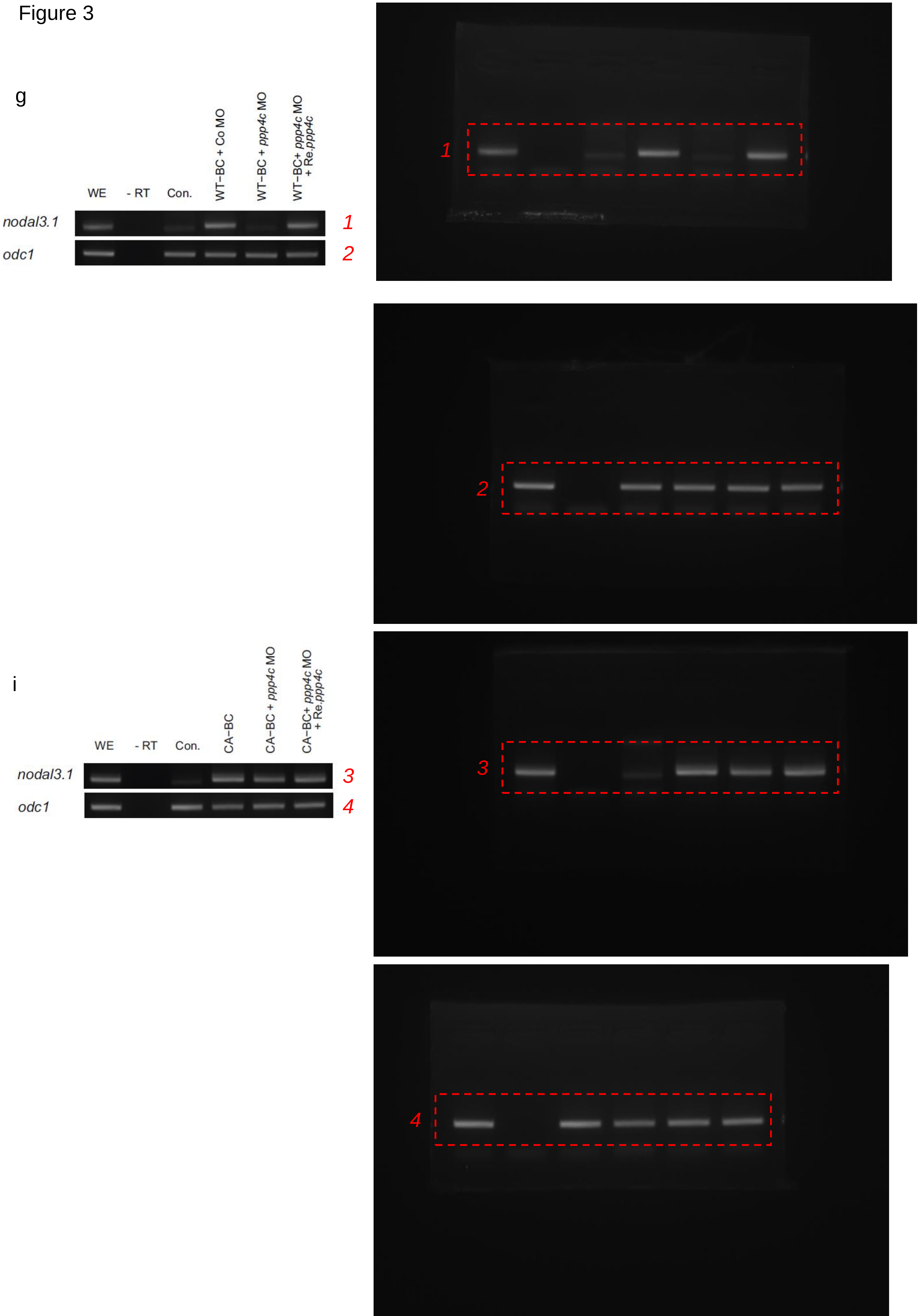

g
WE
- RT
Con.
WT–BC + Co MO
WT–BC + ppp4c MO
WT–BC+ ppp4c MO + Re.ppp4c
nodal3.1
odc1
1
2
i
WE
- RT
Con.
CA–BC
CA–BC + ppp4c MO
CA–BC+ ppp4c MO + Re.ppp4c
nodal3.1
odc1
3
4
1
2
3
4

Figure 4

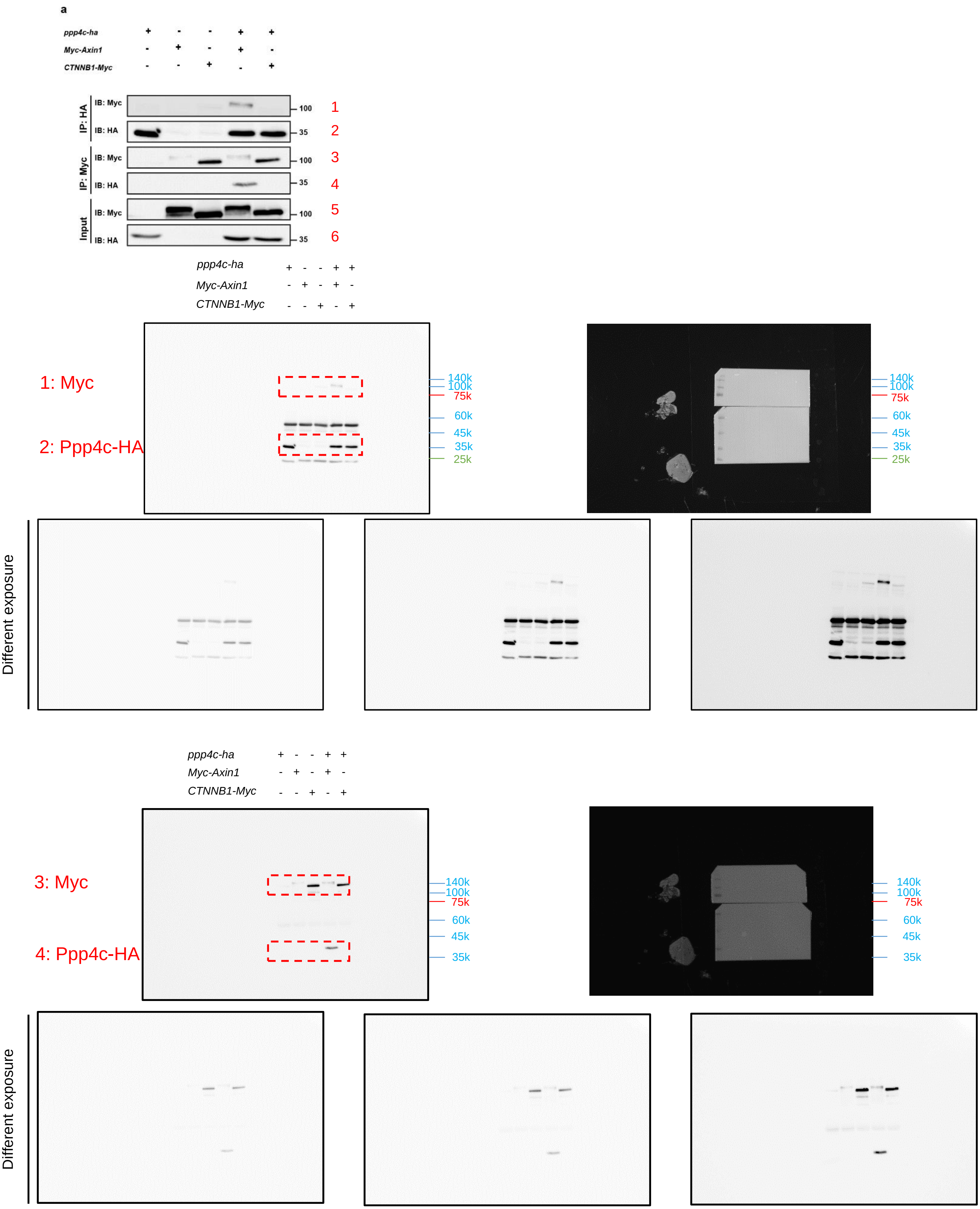

Figure 4a: IP:HA, IP:Myc, Input groups are from different gels, membranes were cropped between 60k-75k. Exposure time: IP: HA, 30 s, IP: Myc, 60 s.

Figure 4

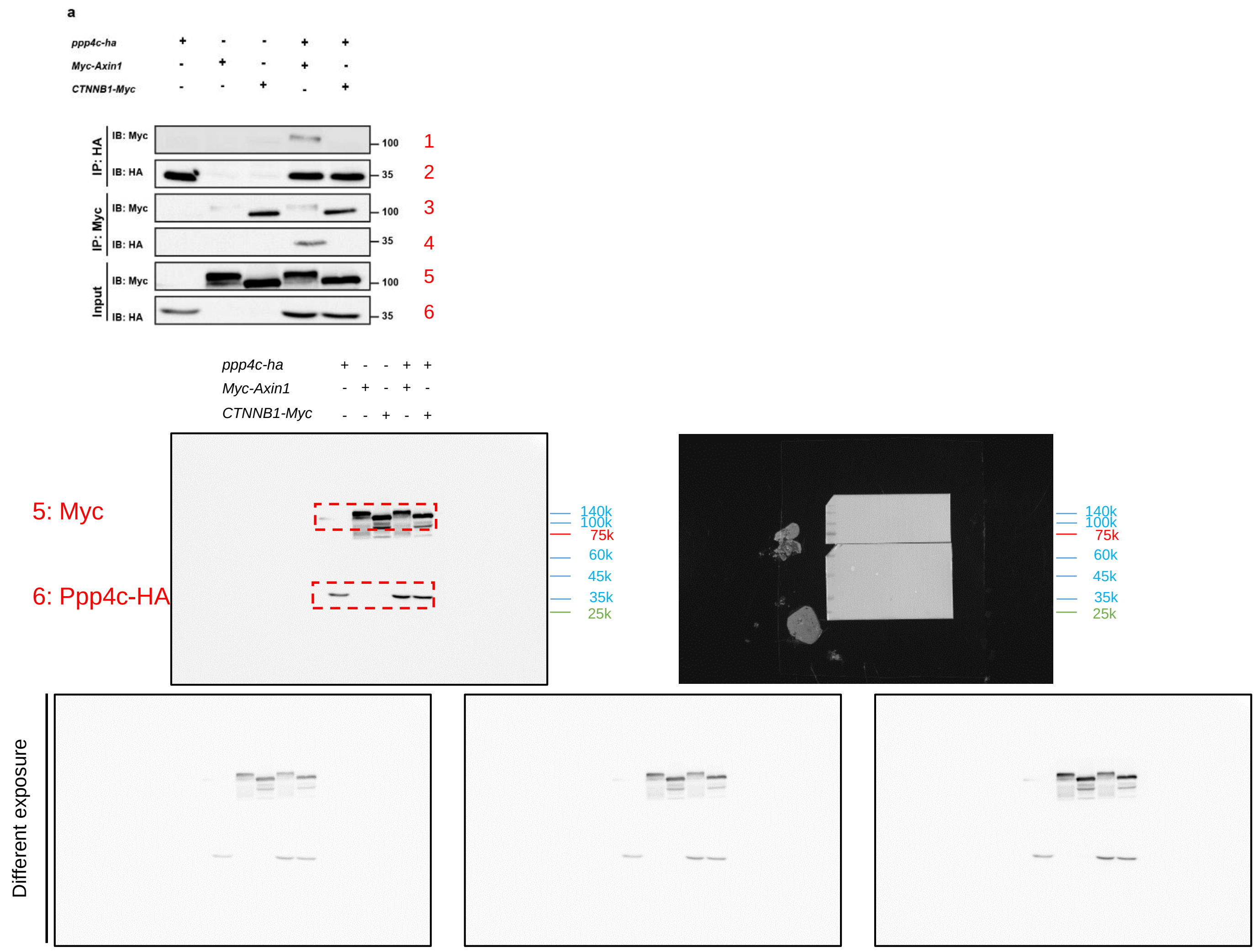

Figure 4a: IP:HA, IP:Myc, Input groups are from different gels, membranes were cropped between 60k-75k. Exposure time: Input: 60 s.

Figure 4

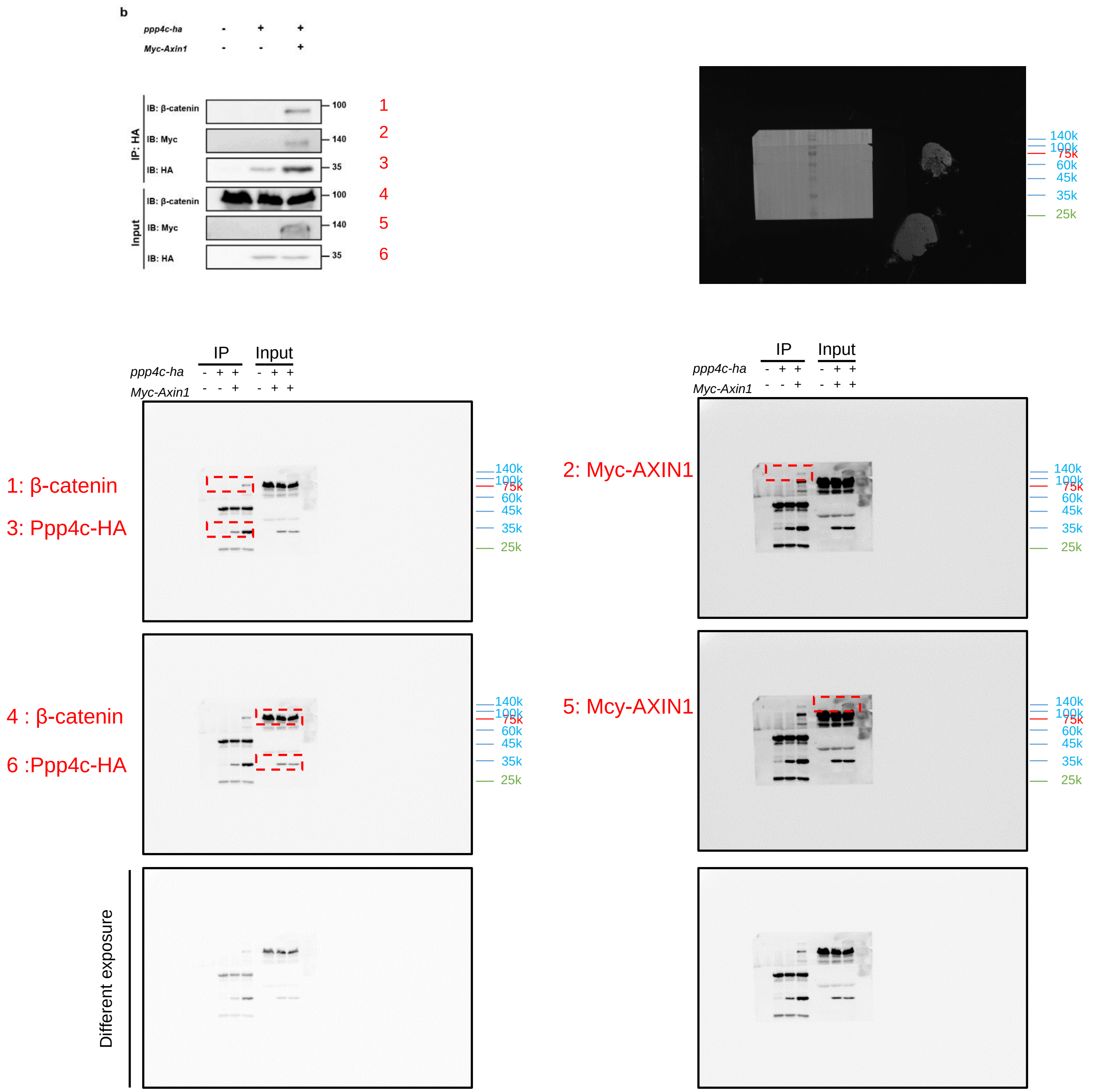

Figure 4b: Same membrane. Membrane was cropped at 60k and 100k. Exposure: Myc, 130 s, else, 70 s.

Figure 4

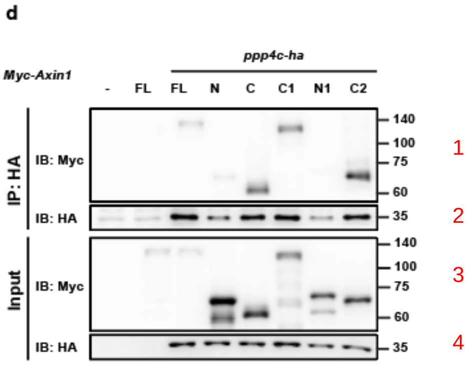

1: Myc

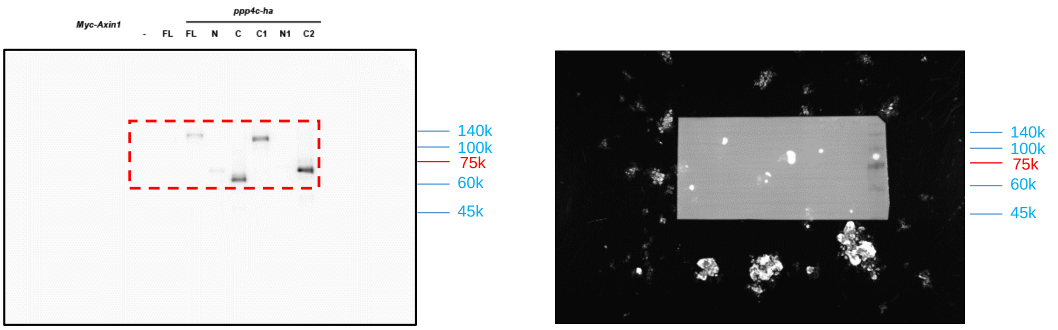

Different exposure

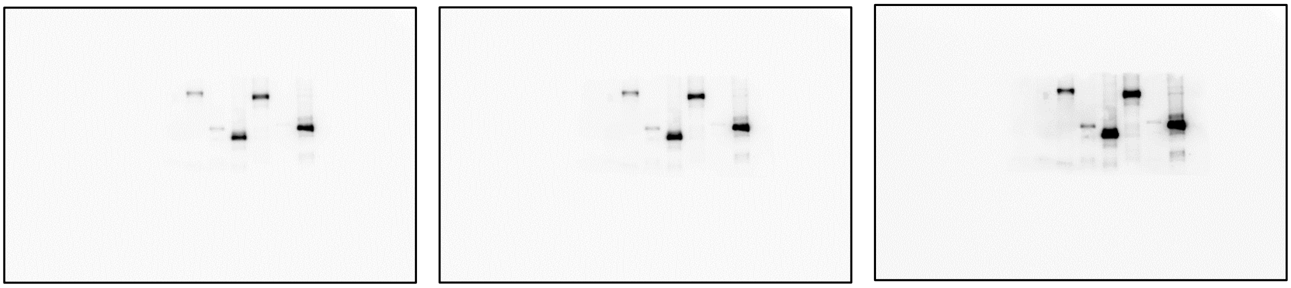

2: Ppp4c-HA

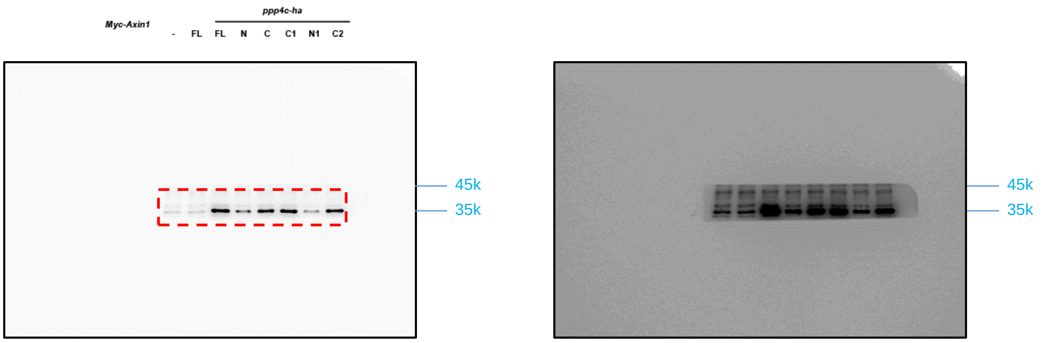

Different exposure

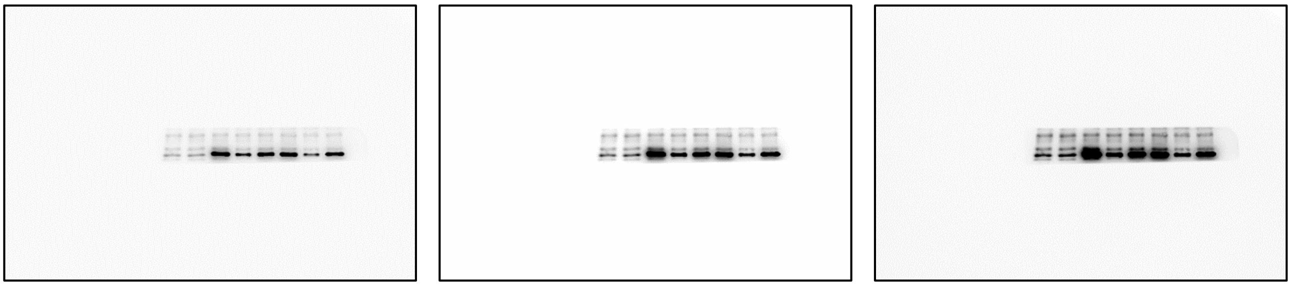

Figure 4d, IP:HA , Input groups are from different membranes. Both membranes were cropped at 45k. Cropped membrane exposed separately, IP-Myc: 10 s, IP-HA: 10 s, Due to ignorance, protein 2-4 failed to snap the markers. The membrane edge was visualized through contrast adjust.

Figure 4

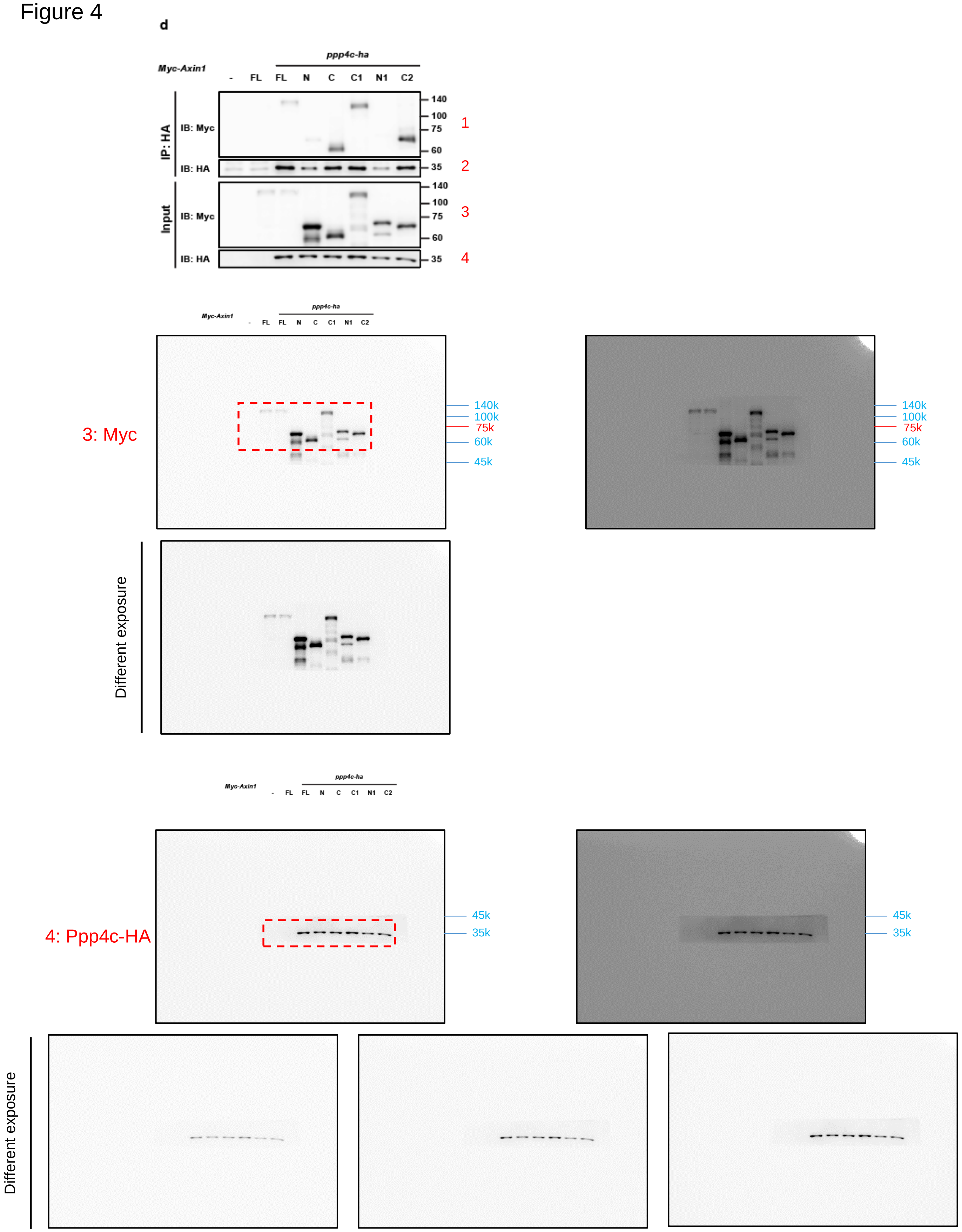

Figure 4d, IP:HA , Input groups are from different membranes. Both membranes were cropped at 45k. Cropped membrane exposed separately, Input-Myc: 10 s, Input-HA: 150 s. Due to ignorance, protein 2-4 failed to snap the markers. The membrane edge was visualized through contrast adjust.

Figure 4

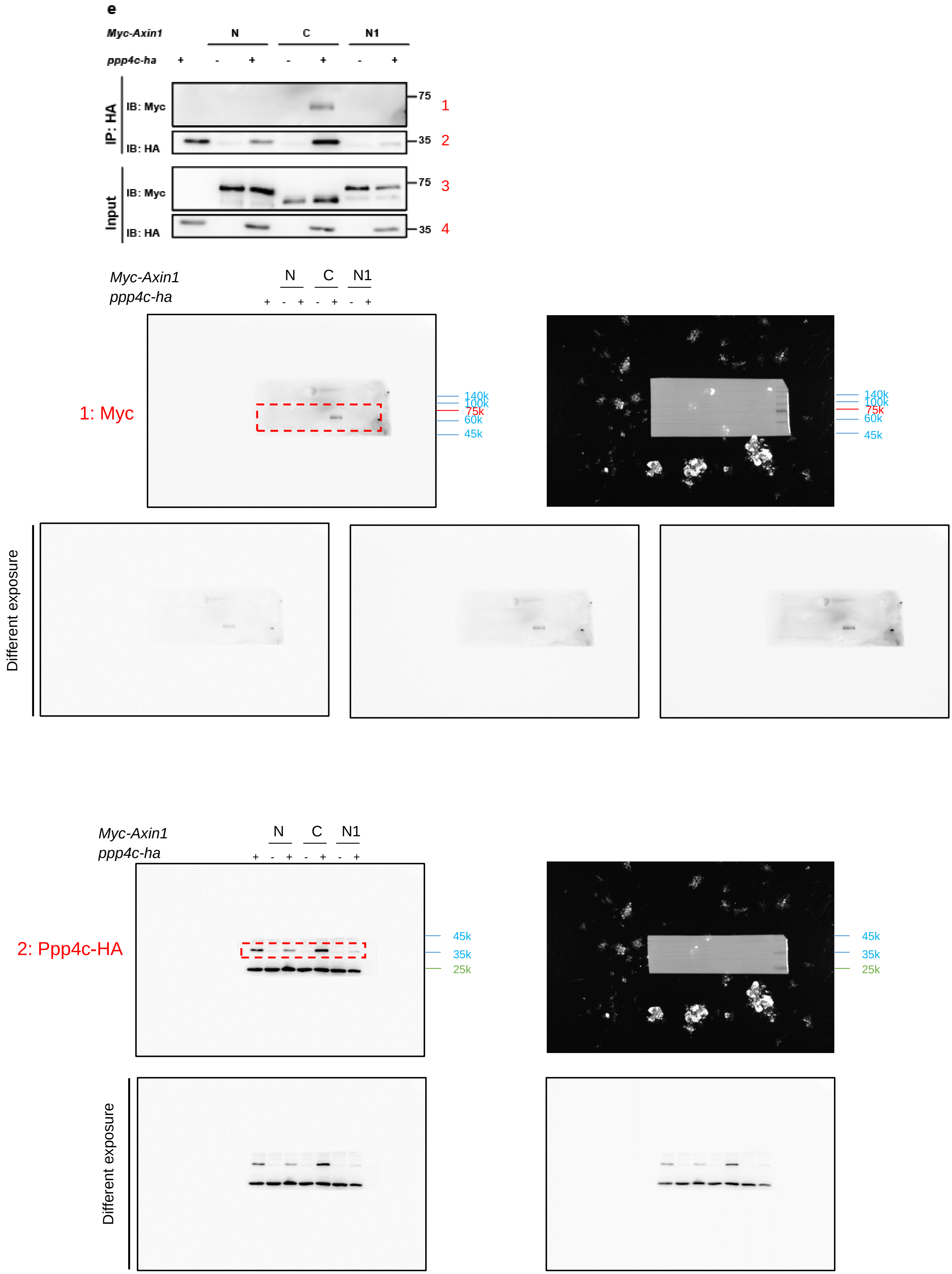

Figure 4e, IP:HA, Input groups are from different membranes. Both membranes were cropped near 45k. Due to the difference between HA and Myc signaling difference, cropped membrane exposed separately (ECL solution), IP-Myc: 110 s, IP-HA: 20 s.

Figure 4

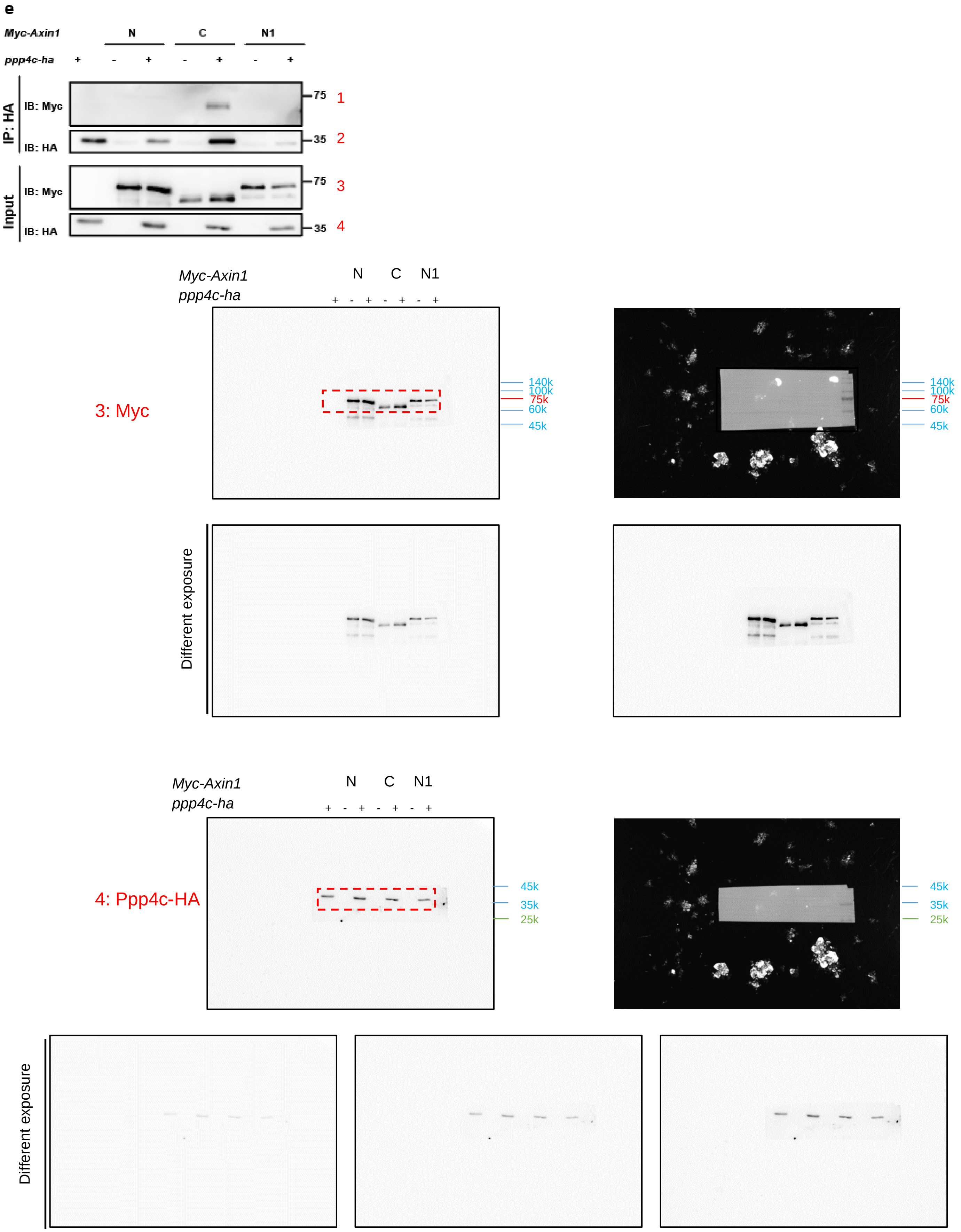

Figure 4e, IP:HA, Input groups are from different membranes. Both membranes were cropped near 45k. Due to the difference between HA and Myc signaling difference, cropped membrane exposed separately (ECL solution), Input-Myc: 20 s, Input-HA: 80 s.

Figure 5

a

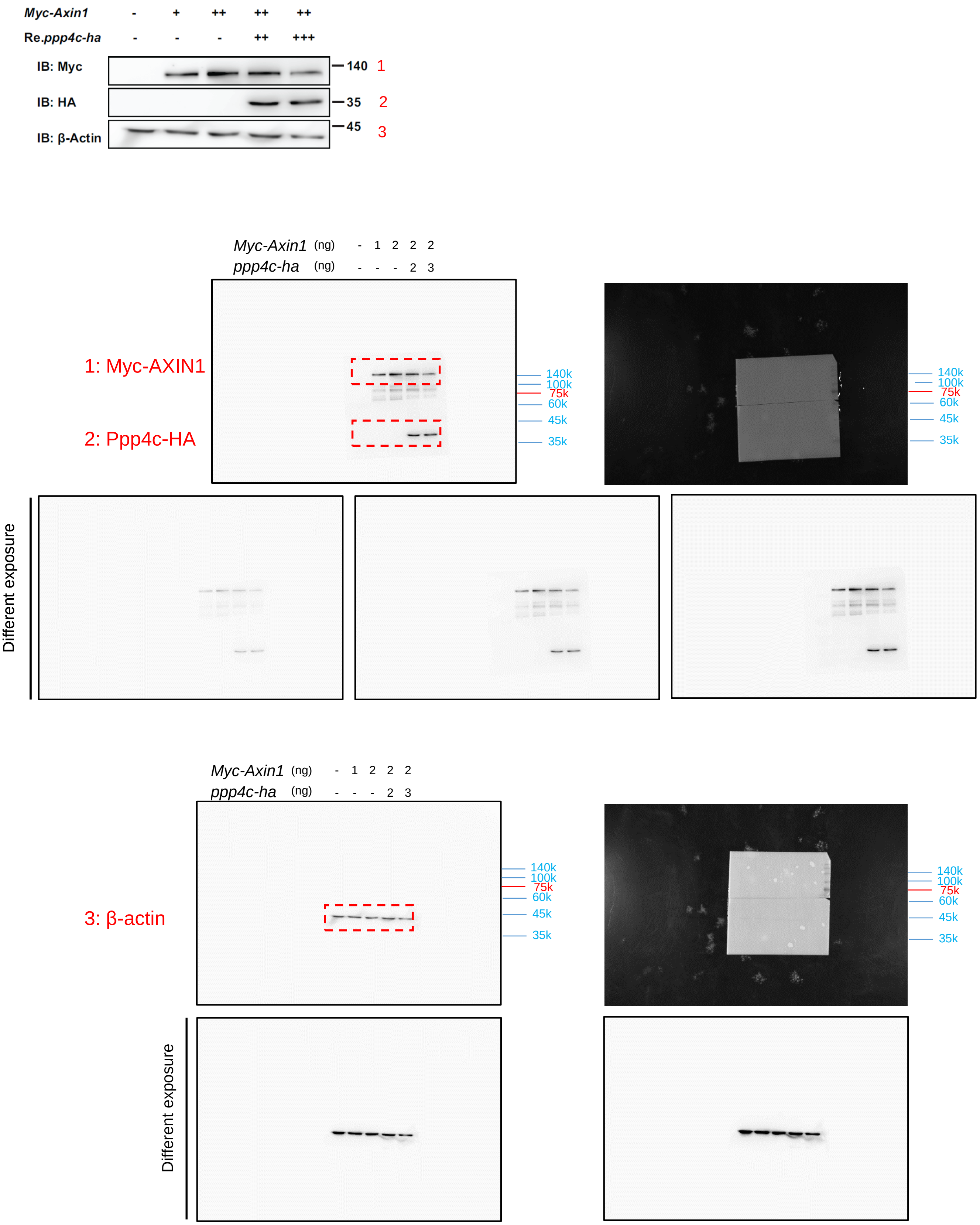

Figure 5a: same membrane, cropped at 60k. Myc: 50 s, HA: 50 s, β-Actin: 5 s.

Figure 5a repeated experiment

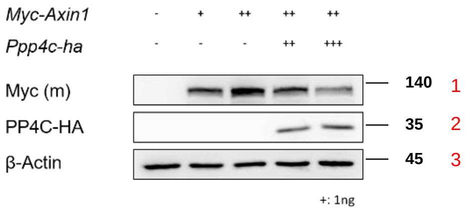

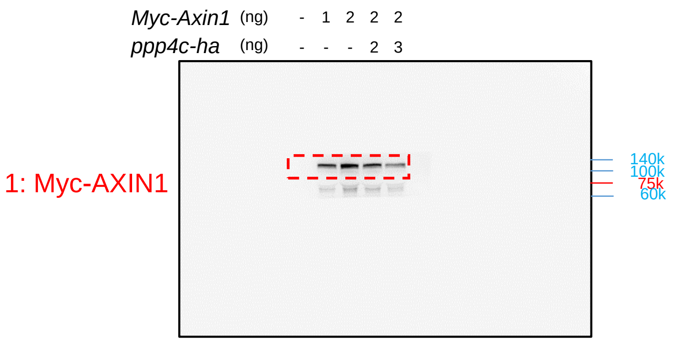

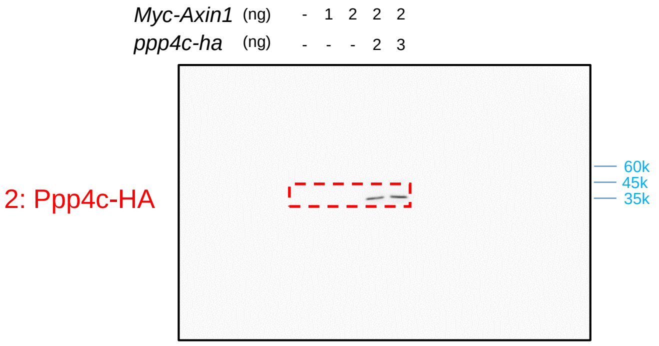

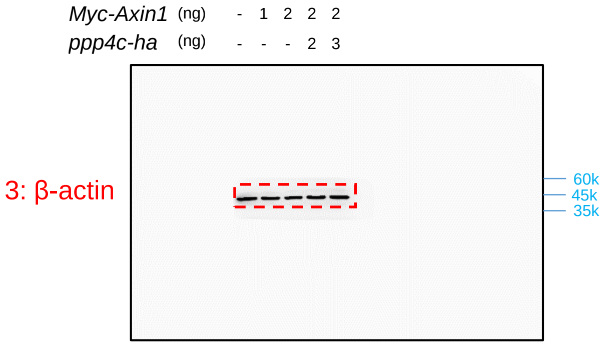

same membrane, cropped at 60k, and exposed seperately. Myc, 5s, HA: 5s, β-Actin: 10 s.

Figure 5b

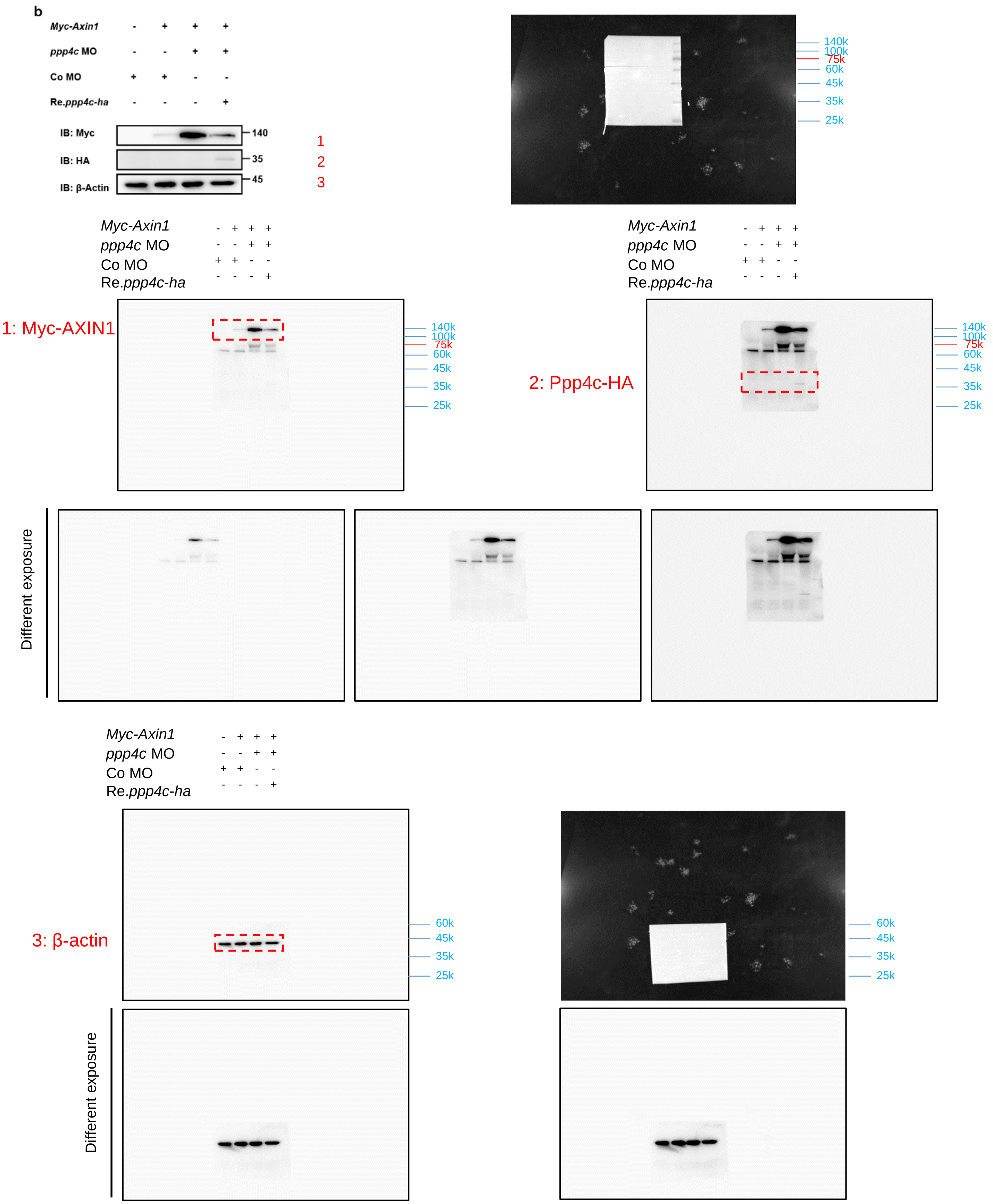

Figure 5b: same membrane, cropped at 60k. Exposure Myc: 30 s, HA: 60 s, β-Actin: 10 s.

Figure 5b repeated experiment

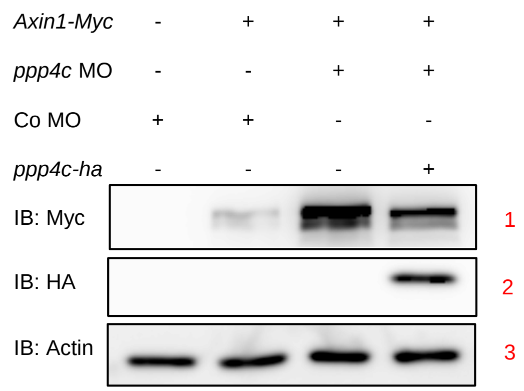

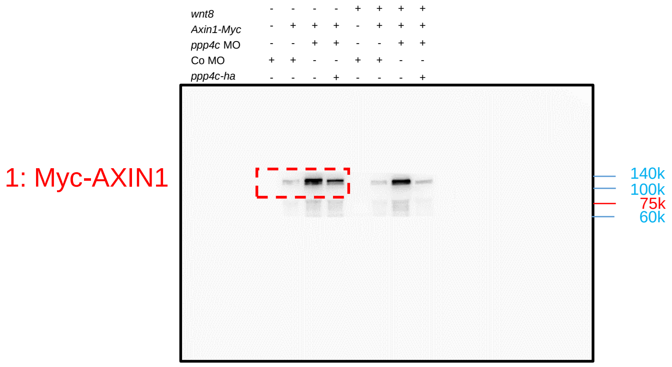

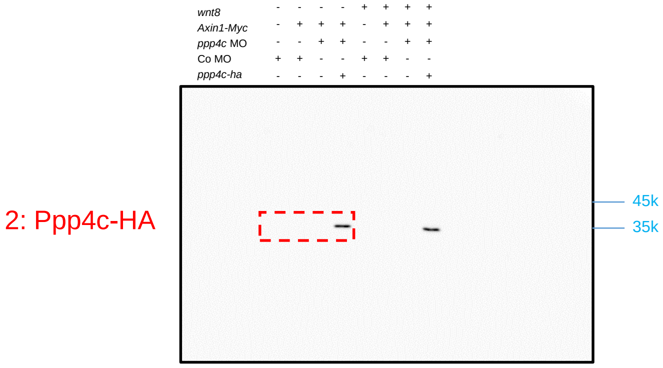

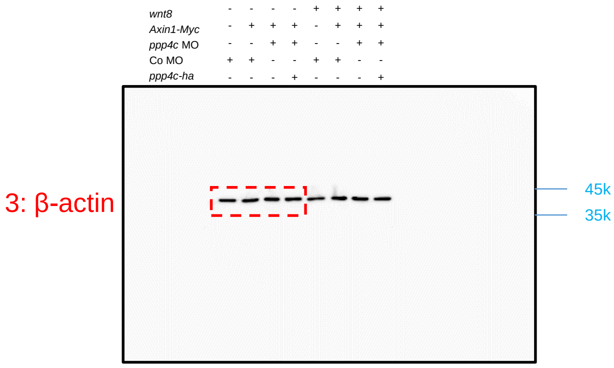

Same membrane, cropped at 45-60k. Exposed separately, Myc, 20s, HA: 40s, β-Actin: 10 s.

Figure 5

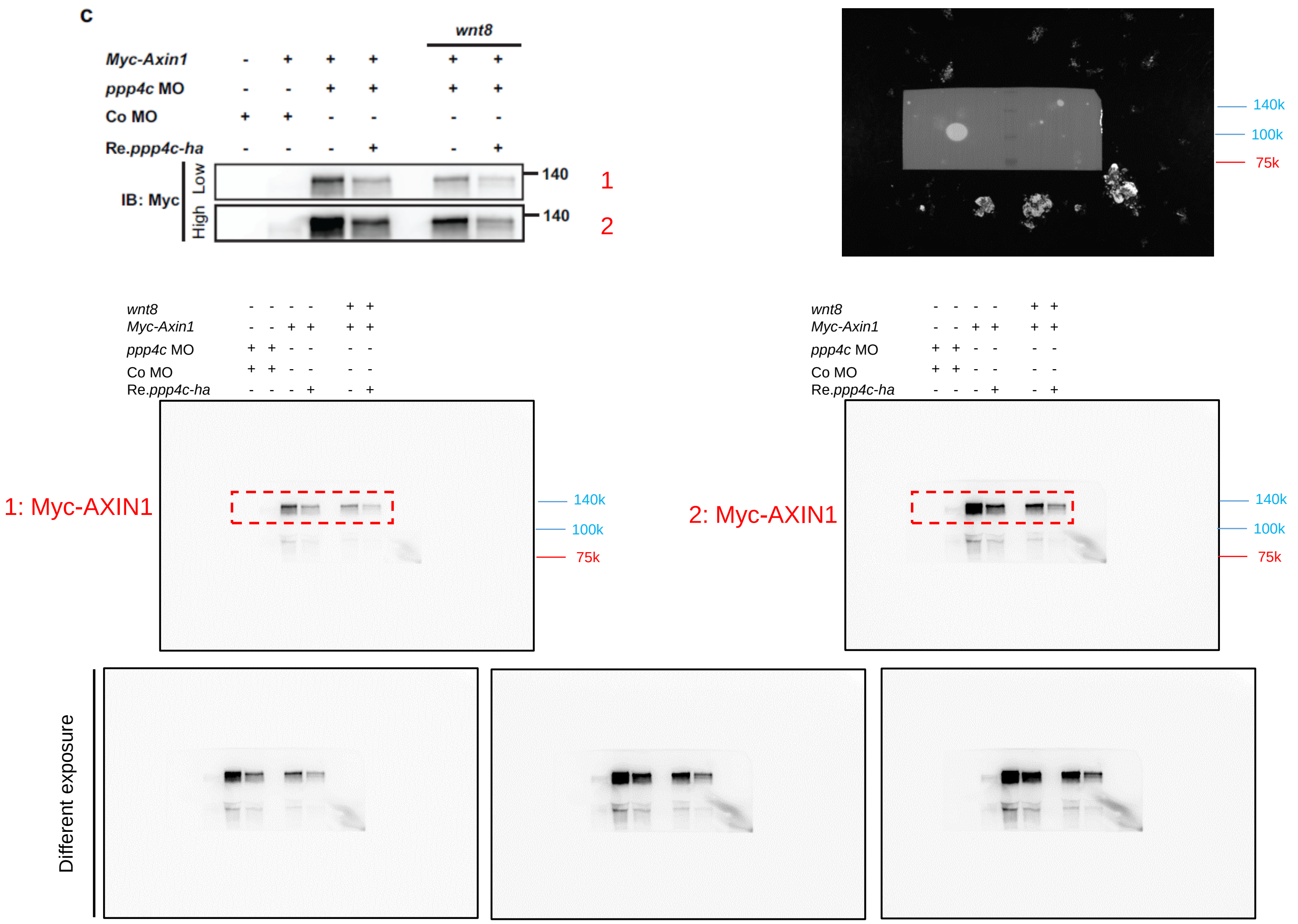

Figure 5c: same membrane, 6.5% gel, exposure low : 20 s, High : 60 s.

Figure 5

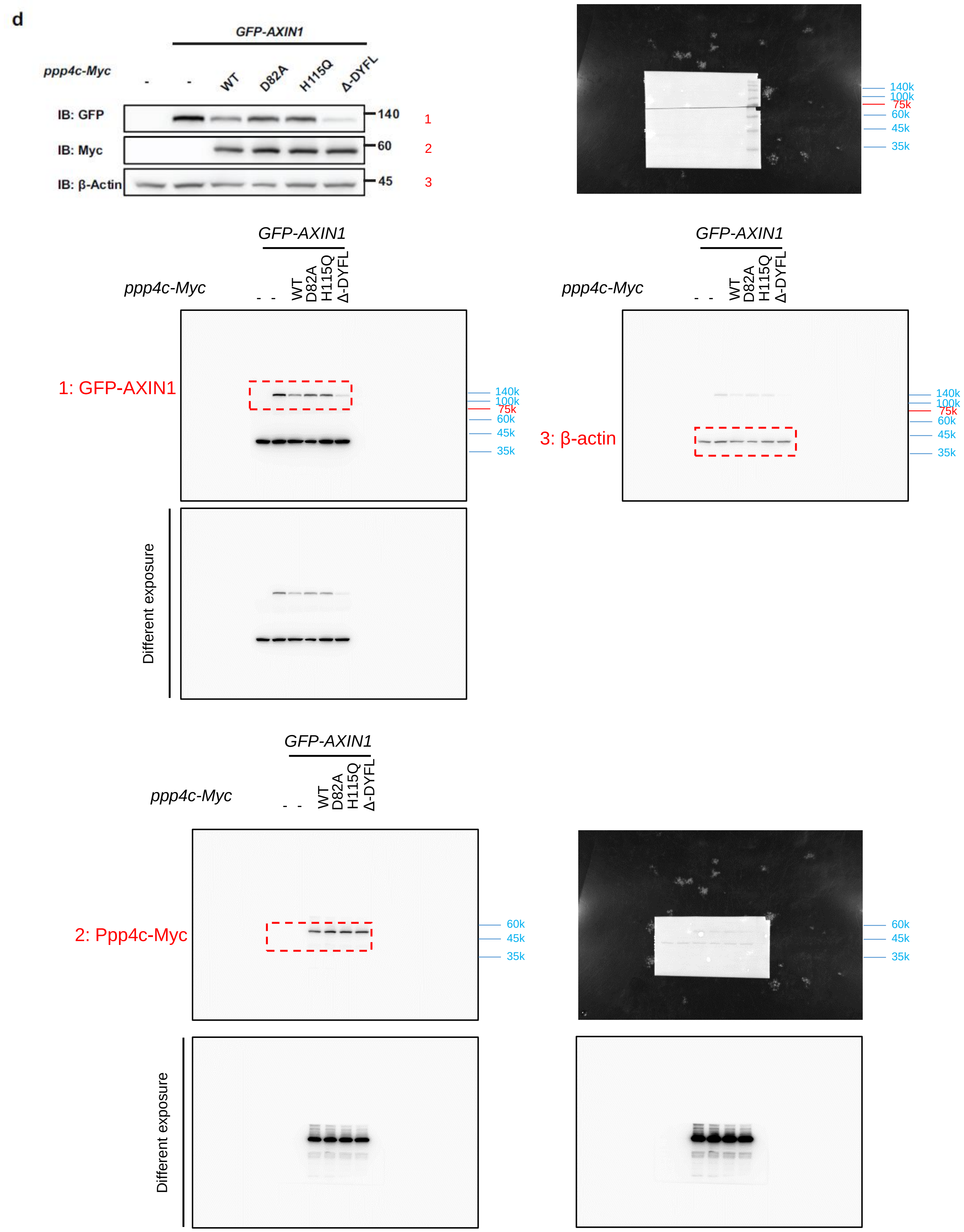

Figure 5d: same membrane, cropped at 75k. Exposure time, GFP: 20 s, Myc: 2 s, β-Actin: 2 s.

Figure 5

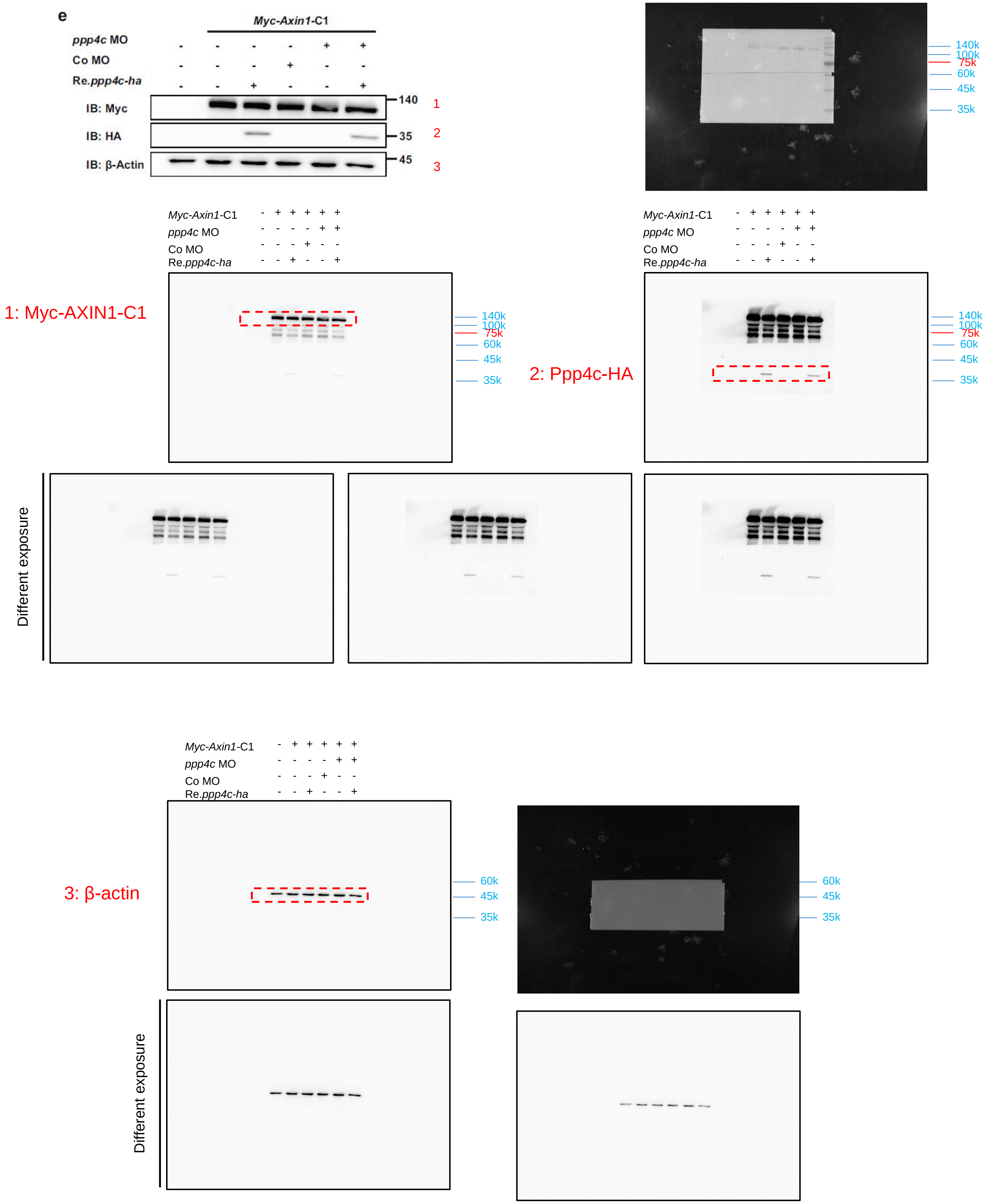

Figure 5e: same membrane, cropped at 60k. Exposure time: Myc, 10 s, HA,70s, β-Actin: 10s.

Figure 5e repeated experiment

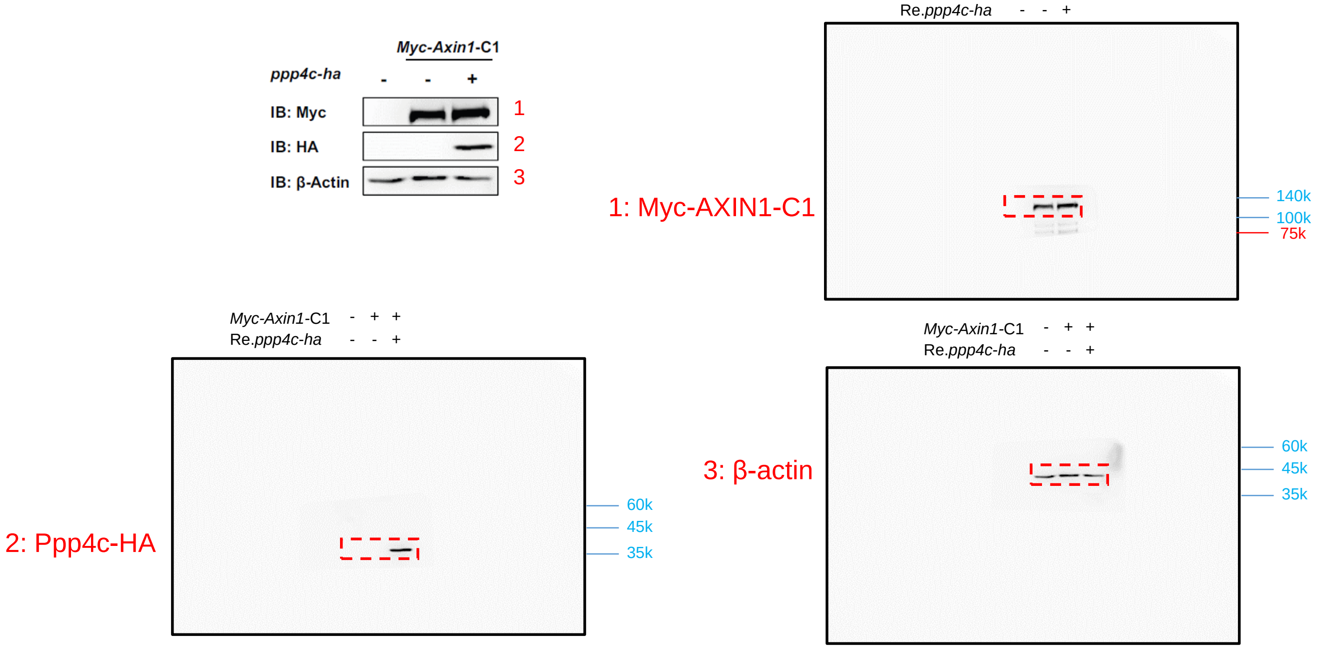

Same membrane, cropped at 60-75k. ECL exposure. Exposed separately, exposure time: Myc, 20 s, HA, 30s, β-Actin: 30s.

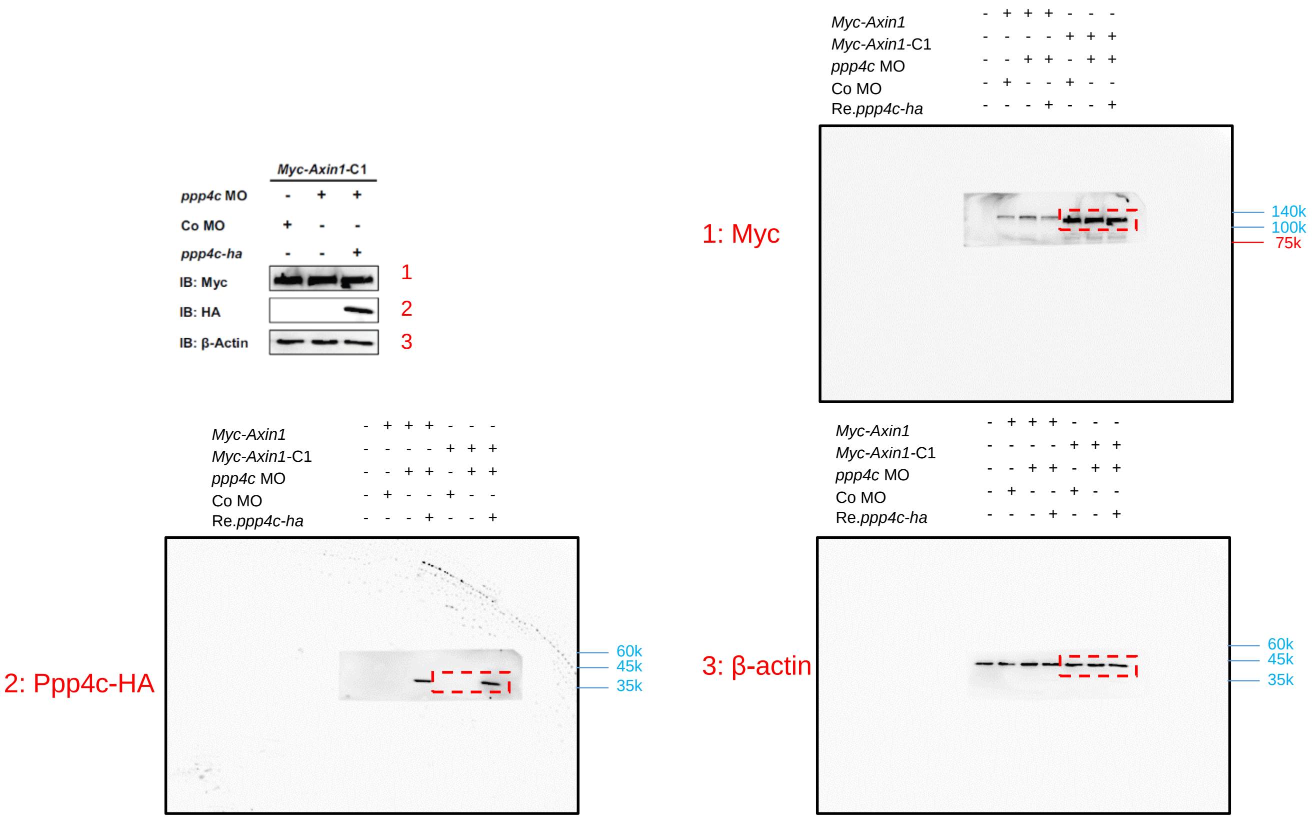

Same membrane, cropped at 60, 75k. ECL exposure. Exposure time: Myc, 10 s, HA, 30s, β-Actin: 30s.

Figure 5

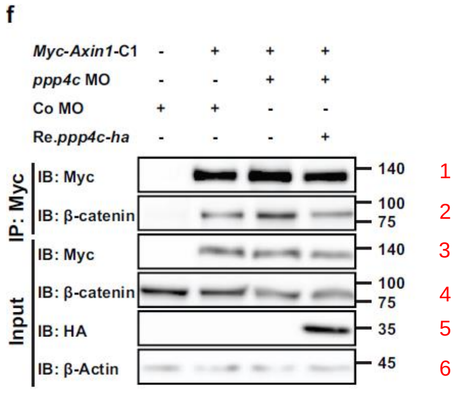

In whole embryo co-IP experiment, due to strong non-specific signaling (Supersignal solution) and the difference between Myc and HA signal (whole membrane blot in repeated experiment-3), the co-IP of whole embryos in Figure 5f were performed using ECL solution for visualization, Input group were cropped at 45 k-60 k before antibody hybridization, and imaged separately.

Repeated experiment - 2

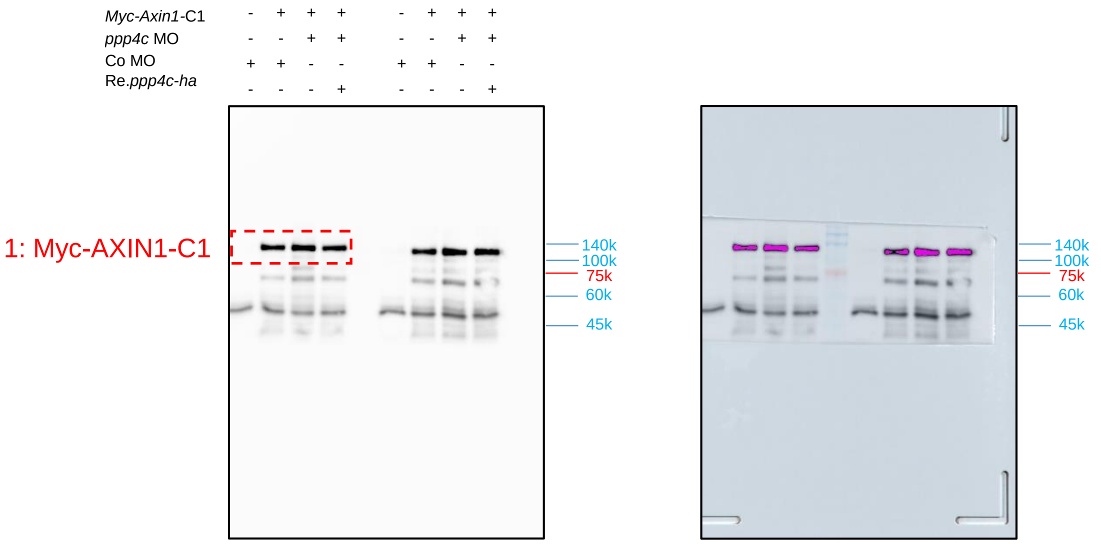

Repeated experiment - 2

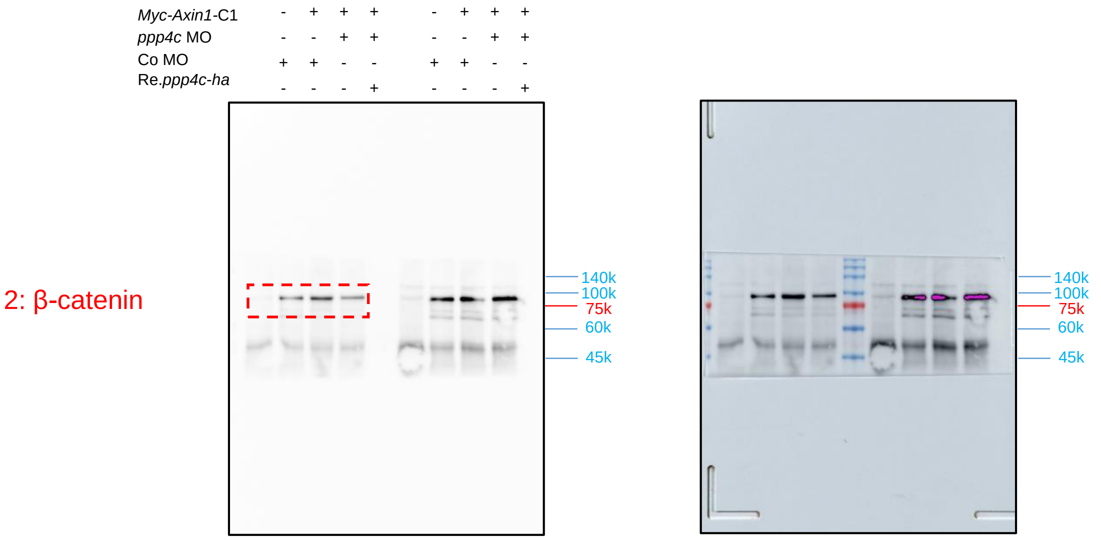

Figure 5f: IP: Myc is from same membrane, exposure ECL solution, exposure time: IP-Myc, 40 s, IP-β-catenin 80 s. Repeated experiment – 2: independent embryo batch.

Figure 5

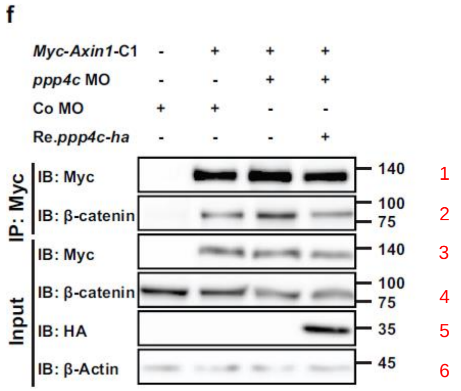

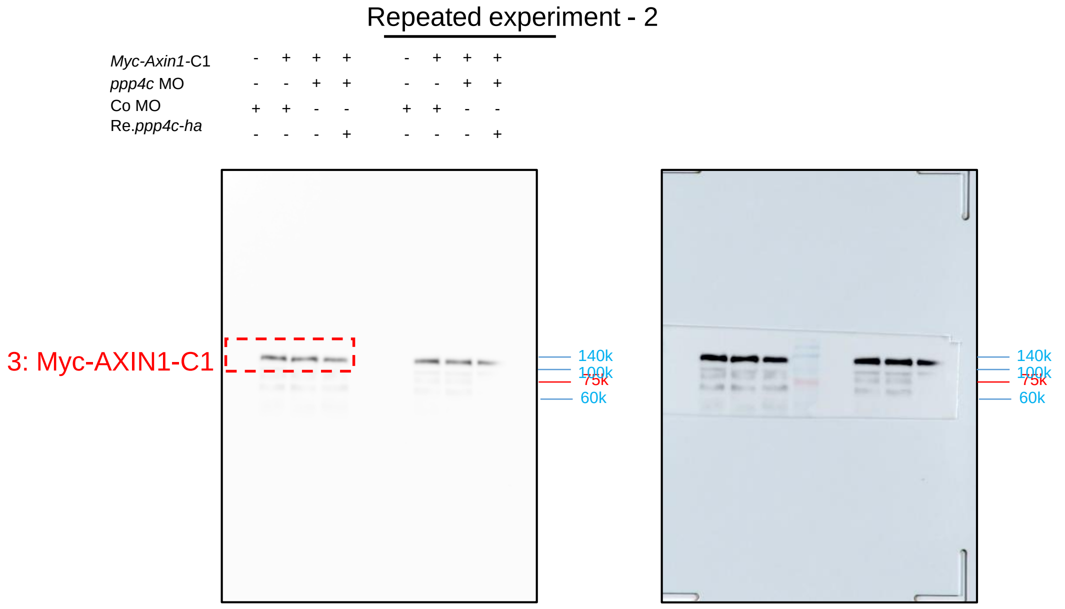

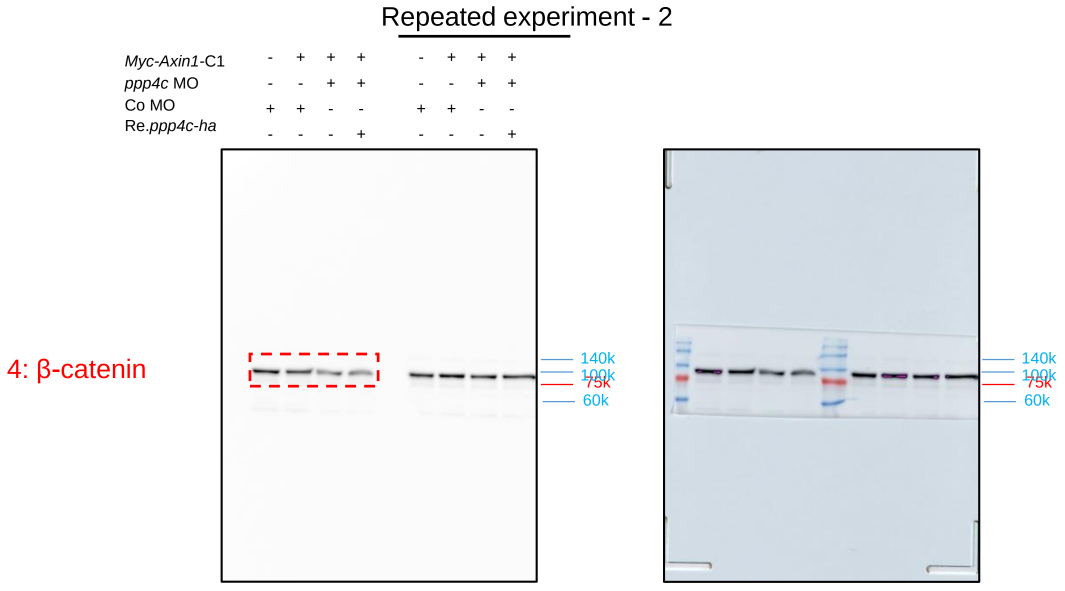

Figure 5f: Input groups is from same gel. Cropped membranes exposed separately (ECL solution), exposure time: Input Axin 80 s, Input β-catenin 90 s, Input HA 40 s, Input Actin 90 s. Repeated experiment – 2: independent embryo batch.

Figure 5

f

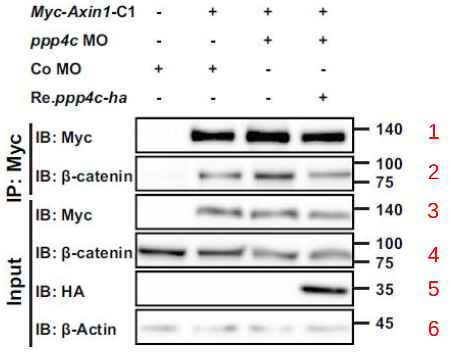

Repeated experiment - 2

Myc-Axin1-C1 - + + + - + + +
ppp4c MO - - + + - - + +
Co MO + + - - + + - -
Re.*ppp4c-ha* - - - + - - - +

5: Ppp4c-HA

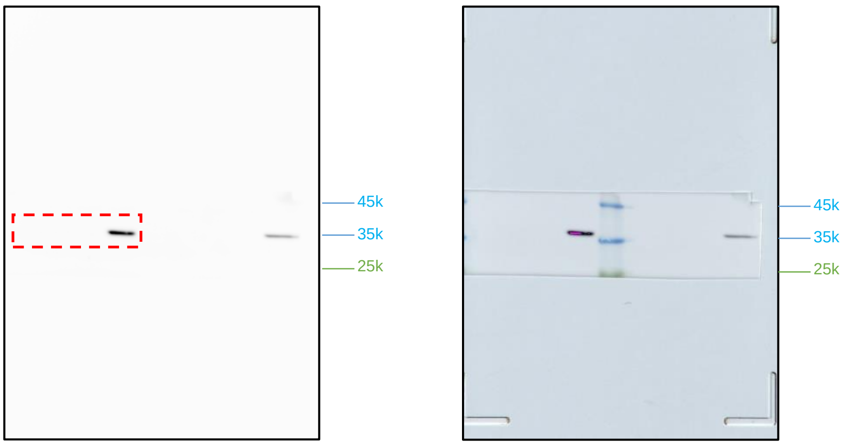

Repeated experiment - 2

Myc-Axin1-C1 - + + + - + + +
ppp4c MO - - + + - - + +
Co MO + + - - + + - -
Re.*ppp4c-ha* - - - + - - - +

6: β-Actin

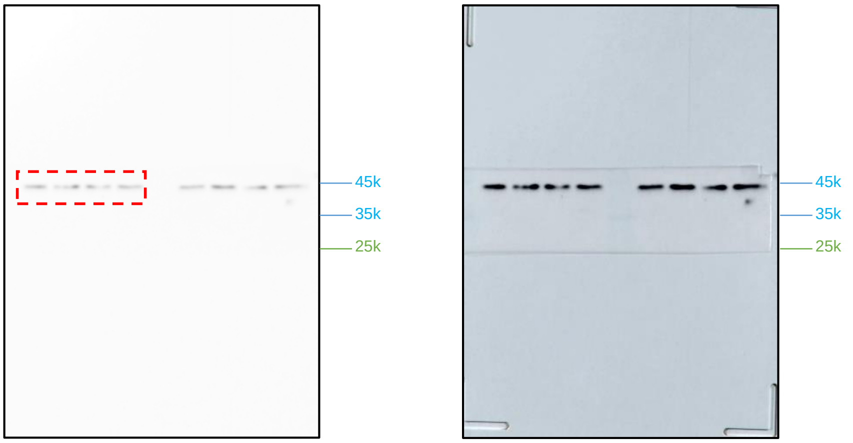

Figure 5f: Input groups is from same gel. Cropped membranes exposed separately (ECL solution), exposure time: Input Axin 80 s, Input β-catenin 90 s, Input HA 40 s, Input Actin 90 s. Repeated experiment – 2: independent embryo batch.

Figure 5f whole membrane blot (Supersignal solution) Repeated experiment - 3

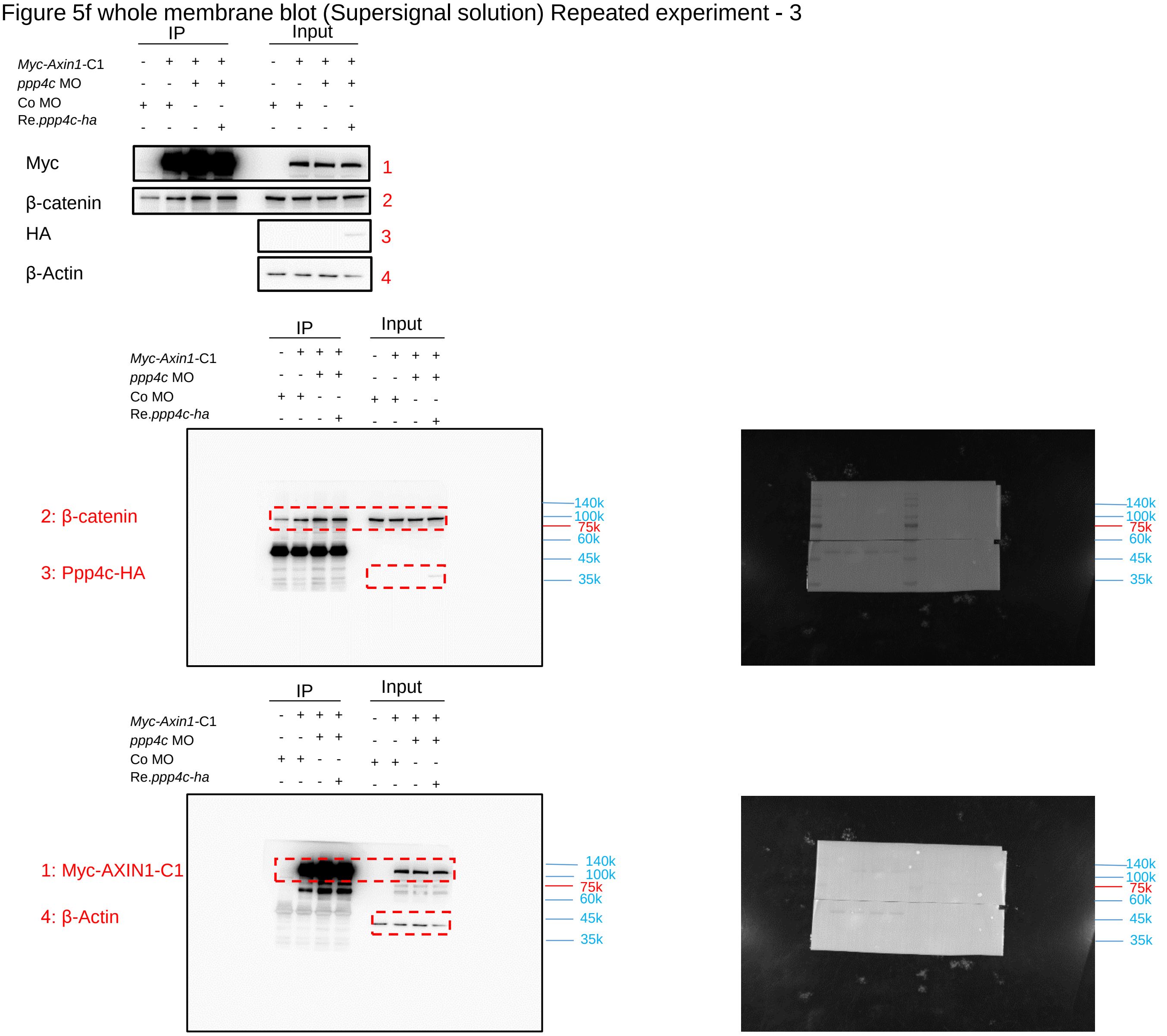

Same membrane. Cropped at 60k. Supersignal solution for visualization, exposure time: Myc, 10 s, β-catenin 40 s, HA 40 s, β-Actin 10 s.

Figure 5f Repeated experiment - 4

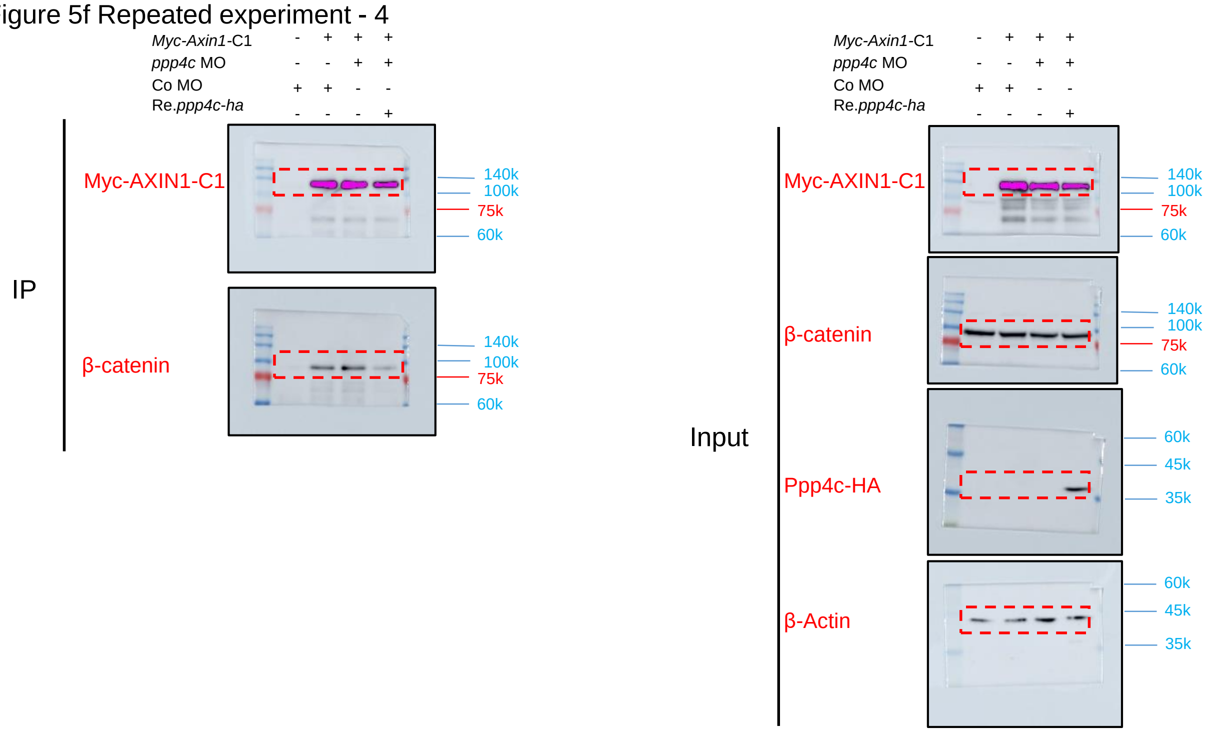

IP: Myc, Input groups are from different gels. Input membrane was cropped at 60k. Cropped membranes exposed separately (ECL solution), exposure time: IP-Myc, 10 s, IP-β-catenin 90 s, Input -Myc 10 s, Input β-catenin 30 s, Input HA 40 s, Input β-Actin 40 s.

SuppFigure 4

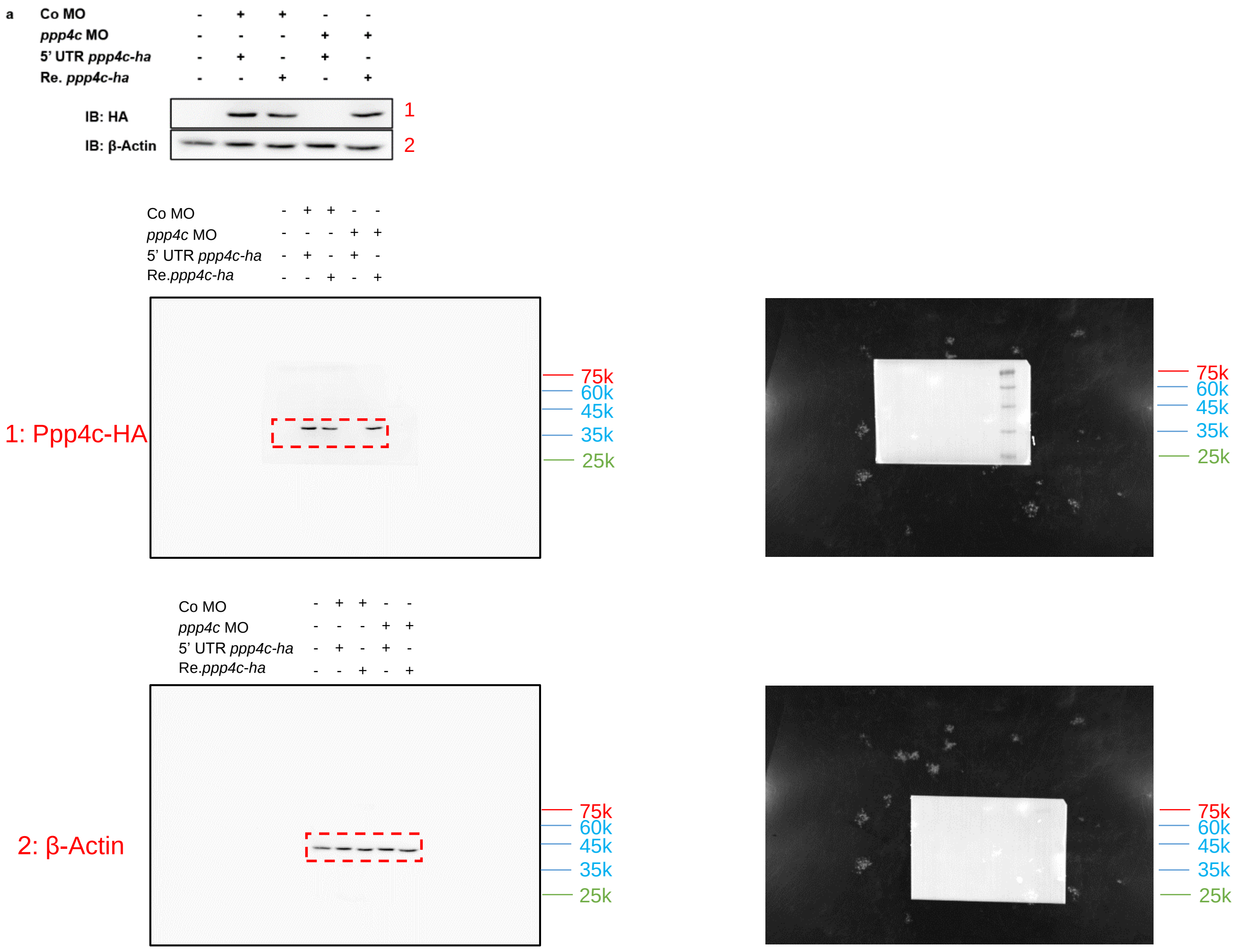

SuppFigure 4a: same membrane, exposure: HA 30 s, β-Actin: 10 s.

SuppFigure 4

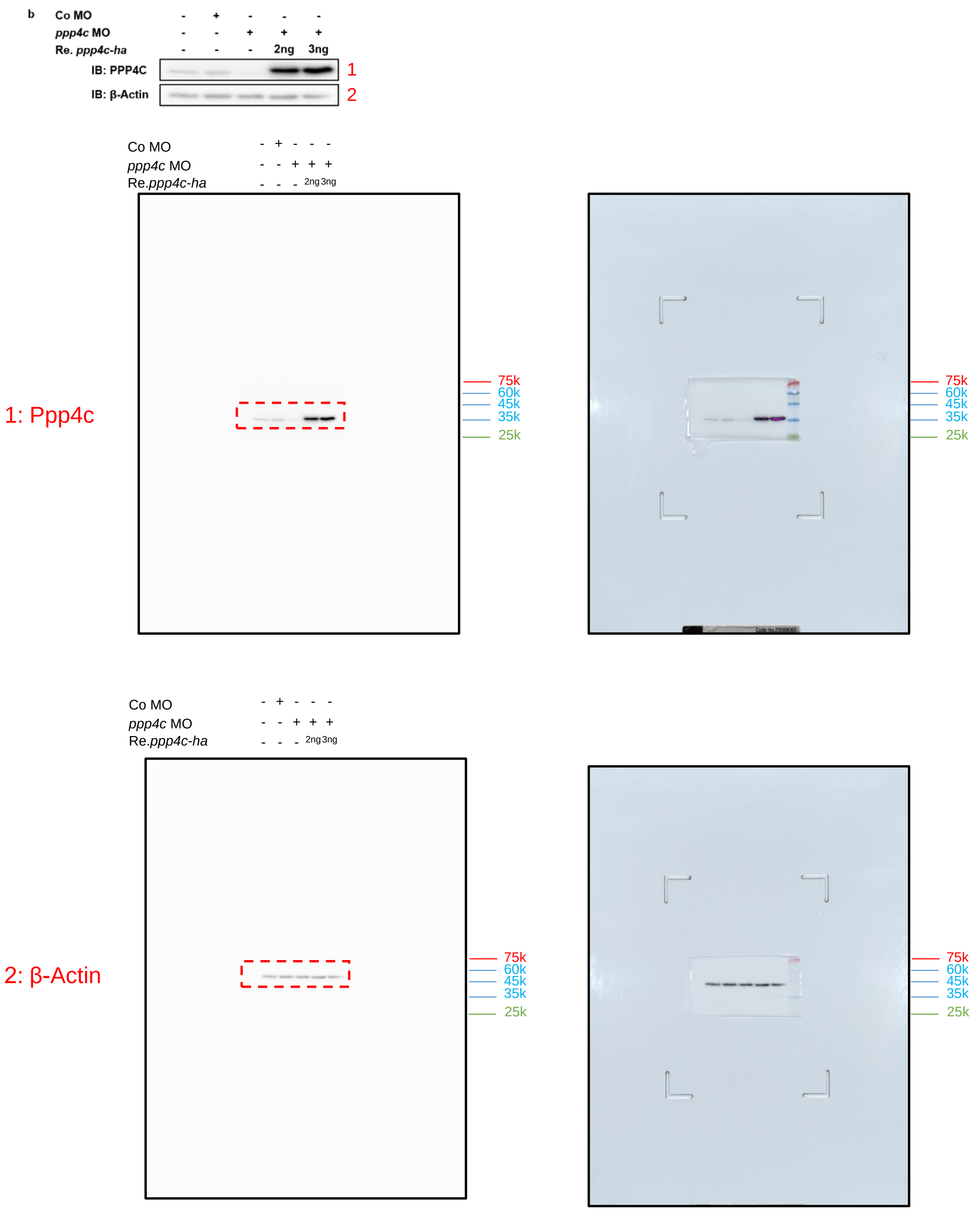

SuppFigure 4b: same membrane, exposure: PPP4C 10 s, β-Actin: 30 s.

Figure 3f, h

	1st				2nd				3rd			
WT-β-catenin Groups	2nd	partial	Normal	Total	2nd	partial	Normal	Total	2nd	partial	Normal	Total
WT-β-catenin 50 pg + Co MO 30 ng	20	7	8	35	18	7	5	30	16	6	10	32
WT-β-catenin 50 pg + *ppp4c* MO 30 ng	8	5	17	30	8	6	17	31	6	6	17	29
WT-β-catenin 50 pg + *ppp4c* MO 30 ng + Re.*ppp4c* 250 pg	22	5	8	35	16	12	7	35	26	2	7	35
CA-β-catenin Groups	2nd	partial	1	Total	2nd	partial	1	Total	2nd	partial	1	Total
CA-β-catenin 20 pg + Co MO 30 ng	26	2	3	31	28	2	2	32	25	4	4	33
CA-β-catenin 20 pg + *ppp4c* MO 30 ng	24	4	5	33	24	1	2	27	21	4	8	33
CA-β-catenin 20 pg + *ppp4c* MO 30 ng + Re.*ppp4c* 250 pg	22	4	4	30	19	10	4	33	23	2	4	29

Figure 6a,b

	1st				2nd				3rd			
Overexpression phenotypes	Normal	Mild	Severe	Total	Normal	Mild	Severe	Total	Normal	Mild	Severe	Total
Control	30	0	0	30	30	0	0	30	30	0	0	30
ppp4c 2 ng	4	19	10	33	7	20	8	35	0	26	6	32
ppp4c 4 ng	0	8	22	30	0	10	21	31	0	9	25	34

Figure 6d,e

	1st				2nd				3rd			
KD-dose phenotypes	Normal	Mild	Severe	Total	Normal	Mild	Severe	Total	Normal	Mild	Severe	Total
Co MO 60 ng	25	0	0	25	30	1	0	31	29	1	0	30
ppp4c MO 30 ng	6	15	29	50	1	19	29	49	5	7	25	37
ppp4c MO 60 ng	1	4	45	50	0	10	40	50	0	6	30	36

Figure 6f,g

	1st			2nd			3rd		
KD+rescue phenotypes	Normal	Defects	Total	Normal	Defects	Total	Normal	Defects	Total
Co MO	24	1	25	26	3	29	30	1	31
ppp4c MO	6	33	39	12	32	44	8	19	27
Rescue	22	12	34	30	17	47	20	8	28

SuppFigure 5

WT-β-catenin (pg)	Complete 2 axis	Partial 2 axis	1 axis	Total
25	9	7	15	31
50	21	5	7	33
100	20	1	8	29
CA-β-catenin (pg)	Complete 2 axis	Partial 2 axis	1 axis	Total
5	0	5	30	35
10	8	10	15	33
20	20	4	6	30
50	21	6	3	30
